# Phenotypic Sorting of Pink Salmon Hatchery Strays May Alleviate Adverse Impacts of Reduced Variation in Fitness‐Associated Traits

**DOI:** 10.1002/ece3.70781

**Published:** 2025-01-08

**Authors:** Julia McMahon, Samuel A. May, Peter S. Rand, Kristen B. Gorman, Megan V. McPhee, Peter A. H. Westley

**Affiliations:** ^1^ College of Fisheries and Ocean Sciences University of Alaska Fairbanks Fairbanks Alaska USA; ^2^ Prince William Sound Science Center Cordova Alaska USA

**Keywords:** dispersal, hatchery–wild interactions, homing, phenology, phenotypic sorting, straying

## Abstract

Maladapted immigrants may reduce wild population productivity and resilience, depending on the degree of fitness mismatch between dispersers and locals. Thus, domesticated individuals escaping into wild populations is a key conservation concern. In Prince William Sound, Alaska, over 700 million pink salmon ( *Oncorhynchus gorbuscha* ) are released annually from hatcheries, providing a natural experiment to characterize the mechanisms underlying impacts to wild populations. Using a dataset of > 200,000 pink salmon sampled from 30 populations over 8 years, we detected significant body size and phenological differences between hatchery‐ and wild‐origin spawners, likely driven by competitive differences during maturation and broodstock selection practices. Variation in traits was reduced in hatchery fish, raising biodiversity concerns. However, phenotypic traits of immigrants and locals were positively correlated. We discuss possible mechanisms that may explain this pattern and how it may reduce adverse impacts associated with reduced trait variation. This study suggests that domestication impacts are likely widespread, but local adaptation may be maintained by phenotypic sorting.

## Graphical abstract

This study investigated phenotypic differences of hatchery‐ and natural‐origin pink salmon in Prince William Sound, Alaska. Analyzing data from over 200,000 salmon, findings indicated significant differences in body size and timing between hatchery and wild fish, with notably reduced variation in these fitness‐associated traits in hatchery fish, raising concerns about reduced biodiversity. However, a positive correlation between the traits of immigrants and locals suggests that phenotypic sorting may help maintain local adaptation despite these impacts.

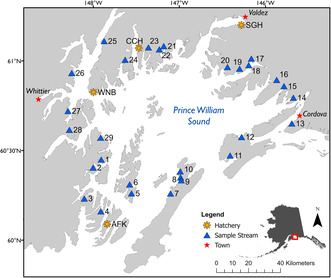

## 1 Introduction

The escape of domesticated animals into natural habitats is an ongoing conservation concern for many taxa, especially those that are highly mobile and predisposed to dispersal (Naish et al. 2008; Bolstad et al. 2021). Domesticated and wild populations can differ in phenotypes associated with Darwinian fitness, raising numerous questions about long‐term ecological and evolutionary consequences, particularly in the context of unintentional introgression of maladapted traits into wild populations (i.e., outbreeding depression; McNeil 1969; Araki et al. 2008; Naish et al. 2008; Willoughby and Christie 2019). However, the impact of domesticated animals interacting with wild populations remains poorly understood in natural systems often because of logistical constraints associated with sampling and identifying domestic and wild individuals on large enough spatiotemporal scales to detect effects (Flagg et al. 2000; Laikre et al. 2010; McMillan et al. 2023). Thus, longitudinal sampling of wild populations at risk of genetic and demographic effects from domestication programs is needed. Intensive sampling programs allow for the quantification of behavioral and phenotypic differences between domesticated and wild populations, which can reveal potential mechanisms leading to introgression and maladaptation and facilitate sustainable management practices for the long‐term viability of wild populations.

One of the large‐scale examples of anthropogenically enhanced populations exists in the Pacific Ocean where approximately 5 billion hatchery‐raised juvenile Pacific salmon (*Oncorhynchus* spp.) are released into the North Pacific Ocean each year (NPAFC 2023). Pacific salmon are known for their anadromous and semelparous life histories, characterized by round‐trip migrations from freshwater juvenile‐rearing habitats, to nutrient‐rich marine environments, then back to natal spawning grounds to reproduce before dying (Quinn 2018). Salmon hatchery practices take advantage of these life‐history strategies to enhance fisheries by rearing juveniles in captivity to circumvent the high mortality that occurs in nature, releasing fish as juveniles into the productive North Pacific Ocean to complete their growth, and harvesting these fish as adults upon their return near the points of release. While this approach has clear potential to increase the total number of returning adults, and therefore harvest opportunity, concerns have been raised about the potential adverse impacts of hatchery‐origin individuals that disperse (termed “hatchery strays” in salmon literature and used henceforth) into wild populations (Flagg et al. 2000; Laikre et al. 2010; McMillan et al. 2023). Importantly, large‐scale hatchery programs can serve as natural experiments for evaluating the ecological and behavioral drivers affecting straying, potentially revealing the mechanisms by which hatcheries may be influencing wild populations (Christie, Ford, and Blouin 2014; Keefer and Caudill 2014; Bett et al. 2017).

Empirical studies of salmon, predominantly of hatchery‐wild systems, have elucidated potential mechanisms underlying salmon straying (Keefer and Caudill 2014). For example, studies have shown how stray rates might be affected by the distances between hatcheries, the ocean, and wild populations (Hard and Heard 1999; Brenner, Moffitt, and Grant 2012; Josephson et al. 2021); life‐history variation such as freshwater residency time, age at return, or size at release from hatcheries (Westley, Quinn, and Dittman 2013; Clarke, Flesher, and Carmichael 2014); stream morphology (Dittman et al. 2010; Cram et al. 2013); or other ecological or climatic factors such as population density and predation risk (Westley et al. 2015; May 2022). In addition, collective migration behavior has been characterized in salmonids, whereby fish move in coordinated groups that can result in groupings of individuals with similar attributes (Berdahl et al. 2016; Westley et al. 2018). Such behavior led us to hypothesize that hatchery strays may spatially sort themselves into wild populations that share similar phenotypes. We characterize this positive spatial or temporal phenotypic covariance between locals and immigrants as “phenotypic sorting”. To our knowledge, patterns of phenotypic sorting have not been formally characterized across metapopulation scales, but such patterns may be expected to emerge from collective or contingent migration behaviors, hatchery effects like broodstock selection practices, or from “matching habitat choice,” whereby individuals assort themselves into physical habitats that best suit their phenotypes, such as the sorting of large‐bodied sockeye salmon ( *O. nerka* ) into deeper stretches of a stream (Camacho and Hendry 2020). Phenotypic sorting also bears similarities to the “favored founders” effect where successful colonizing strays are hypothesized to have traits that are suited to recipient systems (Quinn, Kinnison, and Unwin 2001) or to spatial sorting where assortative mating among dispersive phenotypes can accelerate range expansion (Phillips and Perkins 2019). Yet most studies on salmon straying have been conducted on just one or a handful of populations over limited spatial areas, usually in heavily modified systems, which limits the scale of inference. Large‐scale field studies encompassing many populations over entire watersheds are lacking in the literature, which impedes our ability to characterize metapopulation dispersal processes like phenotypic sorting occurring in nature.

Empirical studies have demonstrated phenotypic differences between hatchery and wild salmon in traits such as body size, return timing, fecundity, and reproductive lifespan (Christie, Ford, and Blouin 2014). These traits are particularly important to study in salmon populations because of their strong correlations with individual fitness (Koch and Narum 2021), underlying genetic variation (Smoker, Gharrett, and Stekoll 1998; Carlson and Seamons 2008), and population productivity (Wertheimer et al. 2004). However, many fitness‐associated traits are genetically and phenotypically correlated, making it difficult to identify the mechanisms driving differences (Thériault et al. 2011). For example, hatchery fish might be larger due to intentional or unintentional selection of larger broodstock or smaller because of unintended effects of rapid growth on maturation (Araki et al. 2008; Berejikian et al. 2012; Milot et al. 2013; McConnell, Westley, and McPhee 2018). Similarly, traits such as instream lifespan and return timing to spawning grounds have been shown to be genetically and phenotypically correlated in salmonids (Hendry et al. 2004; Lin et al. 2016), and these traits are also strongly correlated with reproductive success (Dickerson, Quinn, and Willson 2002; Koch and Narum 2021). Thus, hatchery broodstock selection may change return timing, resulting in mismatches with wild spawning environments, instream lifespans, and fitness (McConnell, Westley, and McPhee 2018; Tillotson et al. 2019). Furthermore, hatchery‐produced salmonids can also exhibit reduced variation in fitness‐associated traits like body size, possibly as a result of hatchery breeding practices (Quinn et al. 2002). Altered natural selection patterns in salmon hatcheries can surface in as little as one to two generations and can be exacerbated by many generations of segregated breeding, possibly leading to decreased variation in fitness‐associated phenotypes and decreased reproductive success when breeding in the wild (Araki et al. 2008; Milot et al. 2013; Christie, Ford, and Blouin 2014; Shedd et al. 2022). However, much of what is known about hatchery‐wild differences comes from spatially limited studies of Chinook ( *O. tshawytscha* ), coho ( *O. kisutch* ), and steelhead ( *O. mykiss* ) populations highlighting a need for examinations of less‐studied species for interspecific comparisons. Therefore, understanding phenotypic differences and underlying mechanisms for specific species and populations is essential for assessing and devising strategies to mitigate potential adverse interactions between hatchery and wild populations and ensure the preservation of both harvest opportunities and ecological integrity.

Among the various salmon species in the North Pacific Ocean, pink salmon ( *O. gorbuscha* ) are by far the most numerous and increasingly appreciated as an important driver of trophic cascades and at‐sea competition (Ruggerone et al. 2023; Rand and Ruggerone 2024). Their strict 2 year life cycle, ease of rearing, and economic significance are qualities that have resulted in pink salmon becoming one of the largest aquaculture programs in the United States (Knudsen et al. 2021; NPAFC 2023; May and Westley 2024). The short life cycle of pink salmon has enabled the study of key demographic and evolutionary trends over many generations (Kovach, Gharrett, and Tallmon 2012; Gharrett, Joyce, and Smoker 2013), which is important for examining the prolonged influences of genetic introgression from hatcheries (Berejikian et al. 2009; Jasper et al. 2013; Shedd et al. 2022). In addition, complete reproductive isolation between sympatric even‐year and odd‐year lineages provides a rare comparative framework for evaluating independent evolutionary processes in replicate habitats (Oke et al. 2019).

Here, we quantify phenotypic differences between hatchery adult pink salmon that have strayed from a large‐scale enhancement program and wild individuals regarding two fitness‐associated traits: Body size (length) and return timing to spawning populations. Our objectives were to (1) examine differences in body size of hatchery and wild fish after accounting for variables documented to affect body size in Pacific salmon (Quinn 2018) such as sex, lineage (even or odd return years), and return timing; 2) determine whether return timing of hatchery and wild fish differed after accounting for variables associated with return timing such as sex, lineage, and body size; and 3) investigate whether straying behaviors were related to body size and run timing. This study is timely given ongoing concerns over the long‐term impacts of hatchery‐origin strays on wild phenotypic and genetic diversity.

## 2 Methods

### 2.1 Study Sites and Data Collection

Prince William Sound (PWS), Alaska, represents the largest pink salmon hatchery program in the world, releasing approximately 600–700 million juvenile pink salmon each year since 1988 (i.e., over 15 generations). Hatchery fish in PWS are spawned randomly from adults that returned to the hatchery (i.e., a predominantly segregated hatchery system). Eggs are hatched in land‐based aquaculture facilities before rearing to net pens for approximately 1 month, after which fry are released to coastal marine environments. Thus, environmental conditions and natural selection are different from wild populations in parent mate choice, incubation, initial feeding, and outmigration, which may underly adaptive or plastic differences between hatchery and wild fish. Hatcheries in PWS use thermal otolith marking, a technique in which controlled temperature changes are applied to fish during early development to create distinctive ring patterns on fish ear stones (Volk, Schroder, and Grimm 1999), to differentiate among hatcheries, release groups (in some instances), brood years, and to distinguish between hatchery and wild‐origin individuals. The unique pink salmon life cycle and large hatchery presence in the region lend itself well to a large‐scale study of divergence between hatchery and wild populations and associated ecological (Ruggerone and Irvine 2018; Knudsen et al. 2021) and evolutionary (Shedd et al. 2022) consequences. Wild pink salmon spawning habitat in PWS occurs in about 1000 coastal freshwater salmon spawning streams terminating in intertidal, marine waters (Giefer and Graziano 2023). Notably, we use the term “wild” to denote fish hatched in natural settings and acknowledge that “wild” streams in PWS have been influenced by hatchery introgression for many generations. In 2011, the Alaska Department of Fish and Game, hatchery industry partners, NGOs, and academia created the Alaska Hatchery Research Program (AHRP) to study hatchery‐origin fish in wild settings. This effort included intensive studies of pink salmon in PWS that demonstrated significant levels of straying in some regions (Knudsen et al. 2021) and, importantly, reduced lifetime reproductive success of hatchery strays spawning in the wild relative to their wild counterparts (Shedd et al. 2022). Here, we used a larger dataset from the AHRP to investigate potential factors that could mechanistically underlie the observed differences in reproductive success between hatchery and wild fish.

**FIGURE 1:**
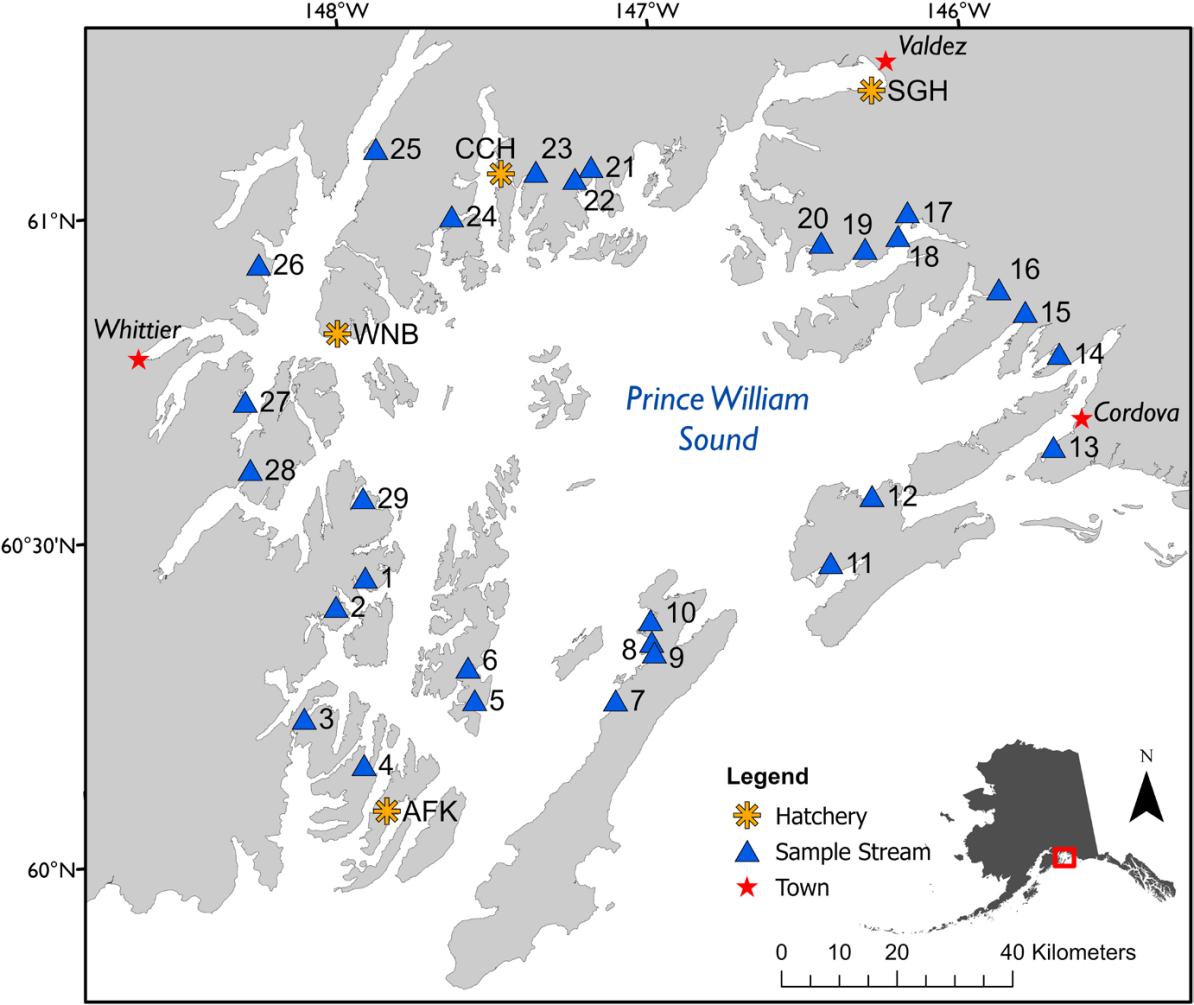
Alaska Hatchery Research Program study populations in PWS, Alaska. Blue triangles mark locations of study streams. Numbered stream names provided in Table S1. Yellow asterisks mark locations of hat cheries that release pink salmon; SGH: Solomon Gulch Hatchery, CCH: Cannery Creek Hatchery, WNB: Wally Noerenberg Hatchery, AFK: Armin F Koernig Hatchery.

Fish were sampled from mid‐July through mid‐September as part of the AHRP from 30 representative stream populations in the PWS, Alaska region between 2013 and 2020 (Figure 1; Table S1). Streams varied in morphology, but most were short (hundreds of meters to several kilometers) and steep, with rain‐dominated hydrology and minimal yet productive salmon spawning habitat, especially in upper intertidal zones. Fine‐scale spatial genetic structure exists in these populations, with many individuals returning to the same areas of the streams where they incubated as eggs and most individuals spawning in the intertidal (Seeb et al. 1999; May et al. 2023). Field efforts focused on achieving representative, random sampling of each population across the spawning season, which in PWS occurs from mid‐July through early September. Stream‐specific details and sample sizes are provided in Table S1.

Field sampling methods have been detailed previously (Knudsen et al. 2021; Shedd et al. 2022). Briefly, fish carcasses were sampled during visual stream surveys throughout the spawning season, and body length from mid‐eye to hypural plate (mm), sex, and sample date (day of year; DOY) were recorded for each individual. Sex was visually identified based on the exaggerated dorsal hump and elongated upper jaw of males when in their spawning morphology, In some cases, gonads were observed directly in the body cavity to confirm sex. Otoliths were excised from all carcasses in the field and later used to determine specific hatchery of origin characterizing individuals as either hatchery (thermally marked) or wild (not marked) by ADF&G (Cordova otolith lab). Fish with body lengths < 200 mm or > 600 mm were excluded from further analyses as these observations were likely data entry errors. Sample date was used as a proxy for return timing, as pink salmon are known to spawn soon after entering spawning habitats (Dickerson, Quinn, and Willson 2002; McMahon 2021).

### 2.2 Examining Differences in Body Length Between Hatchery‐ and Wild‐Origin Adult Pink Salmon

The first objective of this study was to examine whether body size of hatchery‐ and wild‐origin fish differed, after accounting for variables expected to affect body size (i.e., return timing and sex; Shedd et al. 2022). To accomplish this objective, we built a global generalized linear mixed‐effects model (GLMM) with a Gaussian error distribution, where adult body length was the response variable and origin (hatchery or wild), return day, and sex were fixed effects. To allow for sex‐specific intercepts and slopes, interaction terms were included between origin and sex and between return date and sex, which were observed to differ in preliminary analyses. Additionally, population and return year were included as random effects to account for potential population‐ and year‐specific variations in body length. Fish returning in even‐ and odd‐years were modeled separately, as these represent reproductively isolated lineages with known differences in run characteristics and population dynamics (Oke et al. 2019; Knudsen et al. 2021). For all models in this study, continuous variables were scaled to a mean of zero and a standard deviation of one to improve model convergence and comparability between covariates.

Model inference was carried out through a multi‐model selection procedure, applied to all GLMMs in this study. A best‐fit model was determined by exploring a range of simpler models, created by systematically excluding various fixed effects, including comparisons to an intercept‐only null model. Models were compared using a second‐order Akaike Information Criterion, corrected for finite sample size (AICc) to avoid model over‐fitting (Burnham and Anderson 2004). We calculated ΔAICc, the difference between a model's AICc and that of the model with the smallest AICc. All models with ΔAICc ≤ 2 were considered supported models. For a given suite of sub‐models, the influence of fixed effects on response variables was evaluated in the best‐fit model (lowest ΔAICc value).

### 2.3 Examining Differences in Return Timing Between Hatchery‐ and Wild‐Origin Adult Pink Salmon

Similar GLMMs were used to accomplish our second objective, which was to determine if there were differences in the return timing of hatchery‐ and wild‐origin fish. We built a global GLMM with a Gaussian error distribution, where adult return timing (DOY sampled) was the response variable and origin, body length, and sex were fixed effects. As above, interaction terms were included between origin and sex and between body length and sex; population and return year were included as random effects; and even‐ and odd‐year lineages were modeled separately. Pink salmon returning to eastern PWS are known to arrive and spawn up to 2 weeks earlier (approximately mid‐July) than western PWS populations (Helle, Williamson, and Bailey 1964), and this variation was assumed to be accounted for by the random effect of population. As above, model inference was performed using a multimodel selection procedure.

### 2.4 Inference of Phenotypic Sorting of Strays With Regards to Body Length and Return Timing

The final objective of this study was to examine whether the phenotypes of straying hatchery‐origin fish were correlated with the average body size and return timing of recipient wild populations, under the hypothesis that dispersal behaviors of hatchery‐origin individuals are responsive to, and potentially shaped by, the spatial patterns or behavioral cues of wild individuals. We quantified the mean return day and body size of hatchery‐ and wild‐origin individuals within each population and return year. We subtracted these population‐specific values from year‐specific mean return day and body length among all populations, for hatchery‐ and wild‐origin individuals separately. We built two simple global GLMMs with Gaussian error distributions to examine body length and return day of hatchery individuals as response variables, separately. In the first global GLMM, the difference between population‐specific and PWS‐wide mean body lengths of hatchery‐origin individuals was the response variable and was modeled as a function of the difference between population‐specific and PWS‐wide mean body lengths of wild‐origin individuals. Similarly, the second global GLMM examined the difference between population‐specific and PWS‐wide mean sampling date of hatchery‐origin individuals as the response variable, which was modeled as a function of the difference between population‐specific and PWS‐wide mean sampling date of wild‐origin individuals. In both global models, year was included as a random effect to account for interannual variation in body size and return timing, respectively. As in previous models, even‐ and odd‐year lineages were modeled separately. Inference from these models was also based on an AICc model‐selection procedure by comparing each global model to an intercept‐only null model. If the global model was significantly better fit than the null model, we would conclude that wild phenotypes were correlated with the phenotypes of hatchery‐origin fish.

## 3 Results

Our analysis of phenotypic data for adult pink salmon collected from 30 representative populations in the PWS region between 2013 and 2020 included a total of 301,935 fish carcasses, of which 284,867 were pink salmon considered in this study. Otoliths were recovered from 236,413 individuals and analyzed to determine hatchery or wild origin. After removing samples with missing sex data, unreadable otoliths, or length outliers, 218,833 samples remained including 81,674 fish sampled during even‐years and 137,159 fish sampled during odd‐years. Further details on sample sizes by population, year, and origin can be found in the supplemental materials (Table S1).

### 3.1 Examining Differences in Body Length Between Hatchery‐ and Wild‐Origin Adult Pink Salmon

**FIGURE 2:**
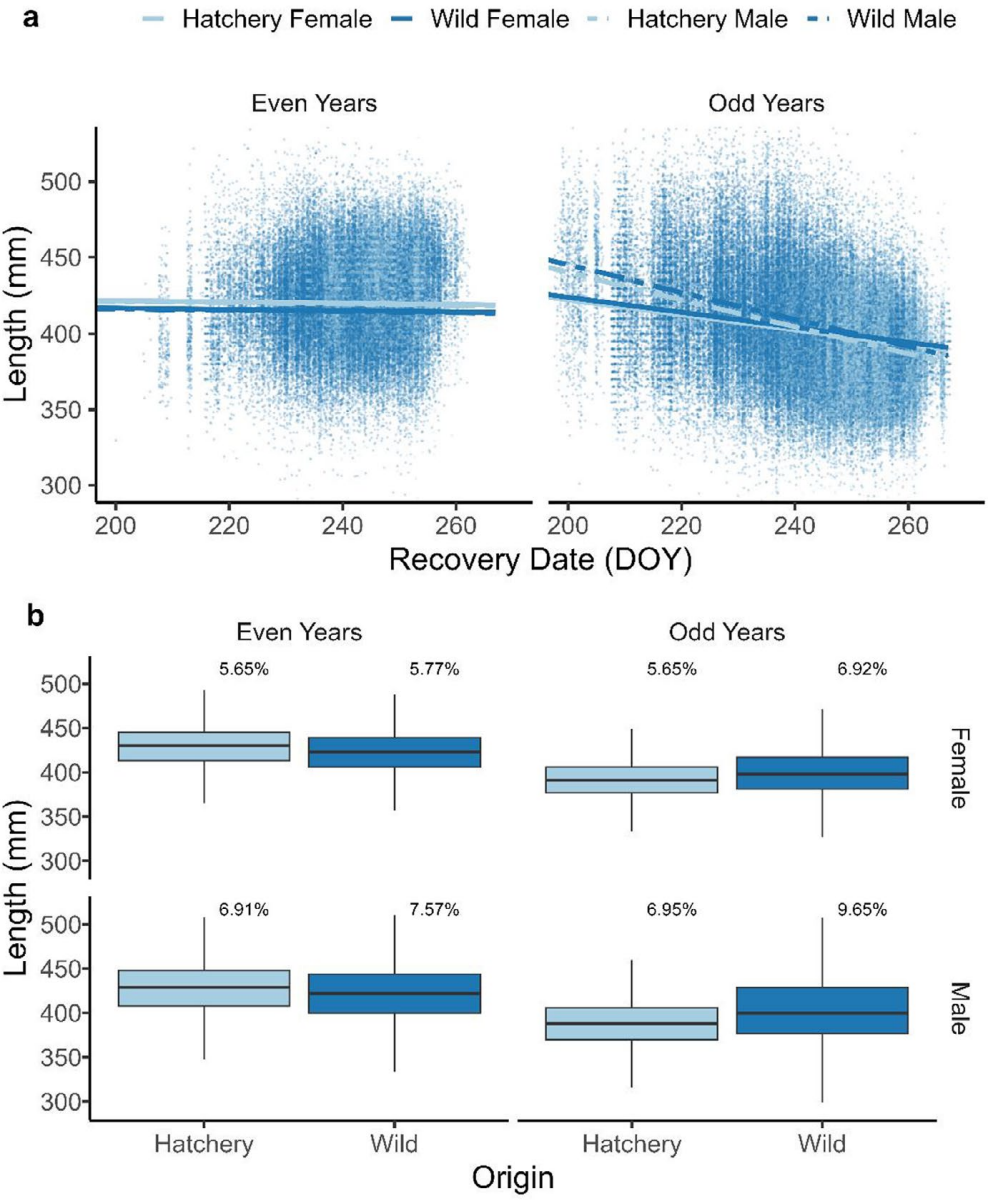
Comparison of body size between hatchery and wild adult pink salmon returning to spawn in Prince William Sound, AK. (a) Regressions of the effects of life history variables on body length (mm). Color hue represents hatchery (light) or wild (dark) origins. Empirical estimates (points) and significant fitted values (lines) are presented for the effect of recovery date (*x*‐axes) on body length (*y*‐axes). Differences between sexes are depicted by solid (female) and dashed (male) model fit lines. Even (left) and odd (right) lineages were modeled separately. To aid visualization, empirical points < 300 mm or > 525 mm were omitted from the plot. (b) Box plots (medians and quantiles; outliers omitted) comparing hatchery‐origin to wild‐origin body size (blues; mid‐eye to hypural length in mm) for each sex and lineage. Inference was performed using generalized linear models (a). Percentages indicate coefficients of variation for the body length of each group.

Models suggested that hatchery fish were larger and smaller than wild‐origin fish in even‐ and odd‐years, respectively (Figure 2). Our best‐fit GLMM investigating body length in even‐years included origin (hatchery or wild), DOY, and sex (*β*_Origin Wild_ = −4.97, 95% CI [−5.46 to −4.48]; *β*_DOY_ = −0.47, 95% CI [−0.74 to −0.20]; *β*_Sex Males_ = −0.59, 95% CI [−0.94 to −0.24]; AICc = 761176.1, *w*AICc = 0.42, *R* ^2^ = 0.24; Table S2). In odd‐years, body length was best explained by the same variables as in even‐years and by additional interaction terms between DOY and sex and between the origin and sex (*β*_Origin Wild_ = 1.71, 95% CI [1.2–2.23]; *β*_DOY_ = −5.63, 95% CI [−5.86 to −5.40]; *β*_Sex Males_ = 0.79, 95% CI [0.23–1.35]; *β*_Wild:Males_ = 3.18, 95% CI [2.49–3.87]; *β*_DOY:Males_ = −4.45, 95% CI [−4.74 to −4.15]; AICc = 1,310,148, *w*AICc = 1.0, *R* ^2^ = 0.22; Table S2). These results indicated that earlier returning fish were larger than later returning fish in both lineages, although this relationship was stronger in odd‐years (Figure 2a). Males were generally larger than females in odd‐years, but females were larger in even‐years (Figure 2b). Interaction terms in odd‐years indicated differences in body size between sexes were smaller in later‐returning individuals than in early returns, potentially with slightly larger females than males in very late individuals (Figure 2a). Boxplots visually demonstrated differences in median and variation in body length among groups defined by variables included in best‐fit models (Figure 2b). Generally, hatchery fish were larger in even‐years and smaller in odd‐years than wild fish, and hatchery strays were less variable than wild fish, as indicated by smaller CV values (Figure 2b) for hatchery strays. Population‐specific boxplots are provided in the supplemental materials (Figure S1).

### 3.2 Examining Differences in Return Timing Between Hatchery‐ and Wild‐Origin Adult Pink Salmon

**FIGURE 3:**
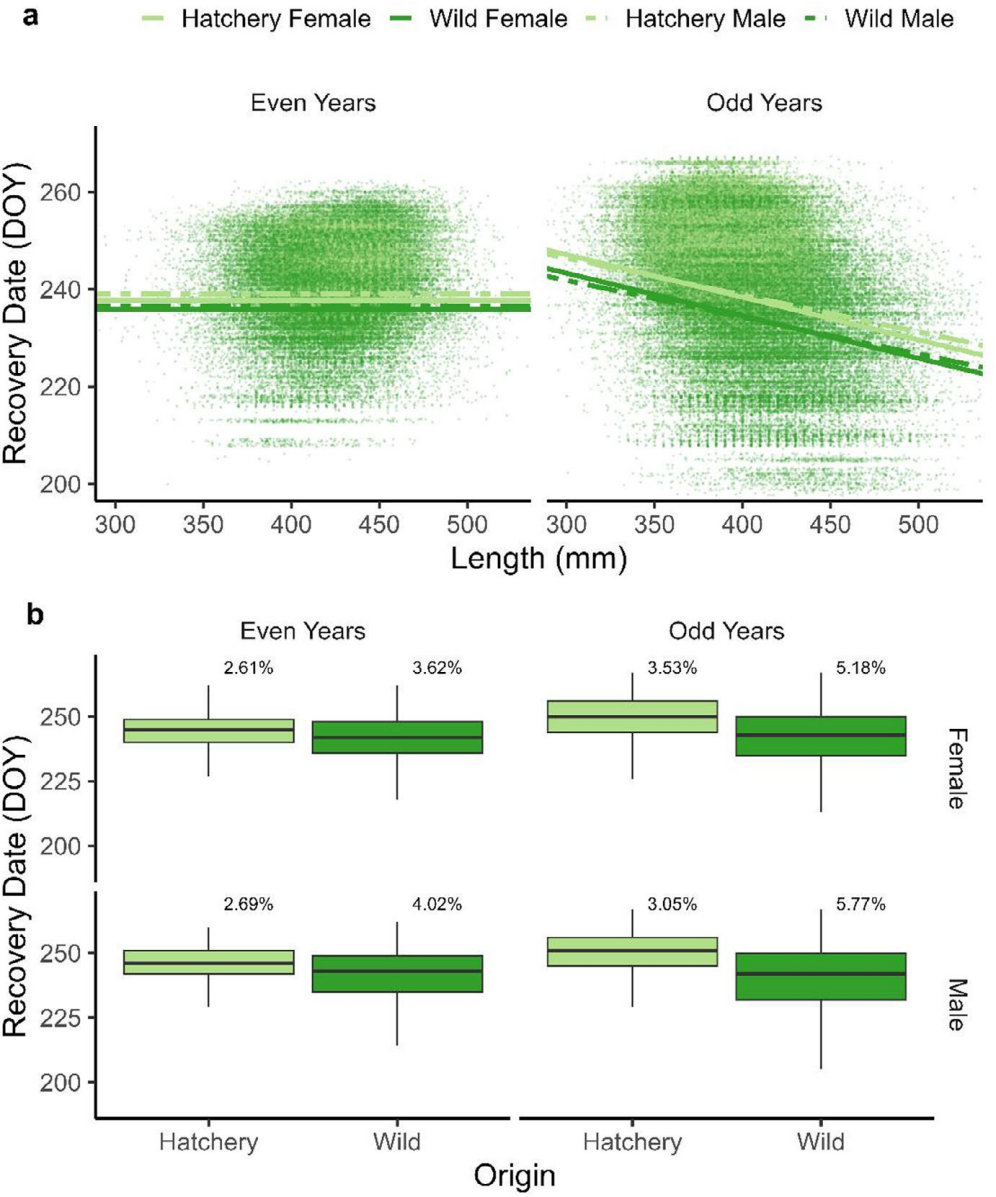
Comparison of recovery date between hatchery and wild adult pink salmon returning to spawn in Prince William Sound, AK. (a) Regressions of the effects of life‐history variables on return timing (day of year sampled, DOY). Color hue represents hatchery (light) or wild (dark) origins. Empirical estimates (points) and significant fitted values (lines) are presented for the effect of body size (*x*‐axes) on recovery date (*y*‐axes). Differences between sexes are depicted by solid (female) and dashed (male) model fit lines. Even (left) and odd (right) lineages were modeled separately. To aid visualization, empirical points < 200 days or > 270 days were omitted from the plot. (b) Box plots (medians and quantiles; outliers omitted) comparing hatchery‐origin to wild‐origin recovery date for each sex and lineage. Inference was performed using GLMs (a). Percentages indicate coefficients of variation for the recovery date of each group.

Hatchery fish returned later than wild‐origin fish in both even and odd lineages (Figure 3). The best‐fit GLMM examining return timing in even‐years included origin, sex, and an interaction between these two covariates (*β*_Origin Wild_ = −0.16, 95% CI [−0.18 to −0.15]; *β*_Sex Males_ = 0.11, 95% CI [0.09–0.13]; *β*_Wild:Males_ = −0.06, 95% CI [−0.09 to −0.04]; AICc = 159,688, *w*AICc = 0.96, *R* ^2^ = 0.59; Table S2). In odd‐years, return timing was best explained by length, origin, sex and interaction terms between length and sex and between origin and sex (*β*_Origin Wild_ = −0.34, 95% CI [−0.35 to −0.32], *β*_Length_ = −0.01, 95% CI [−0.01 to −0.01]; *β*_Sex Males_ = −0.37, 95% CI [−0.49 to −0.25]; *β*_Wild:Males_ = −0.07, 95% CI [−0.09 to −0.05]; *β*_Length:Males_ = 0.001, 95% CI [0.001–0.001]; AICc = 346343.5, *w*AICc = 1.0, *R* ^2^ = 0.46; Table S2). From these results, we concluded that females returned earlier than males in even‐years, but in odd‐years, there were pairwise interactions between sex, return timing, and body size, indicating that small males returned earlier than small females, but large males returned later than large females (Figure 3a). As with body length, hatchery fish were notably less variable in return timing than wild fish, as indicated by reduced CVs in all groups (Figure 3b). Population‐specific boxplots of return day are provided in the supplemental materials (Figure S2).

### 3.3 Inference of Phenotypic Sorting of Strays With Regards to Body Length and Return Timing

**FIGURE 4:**
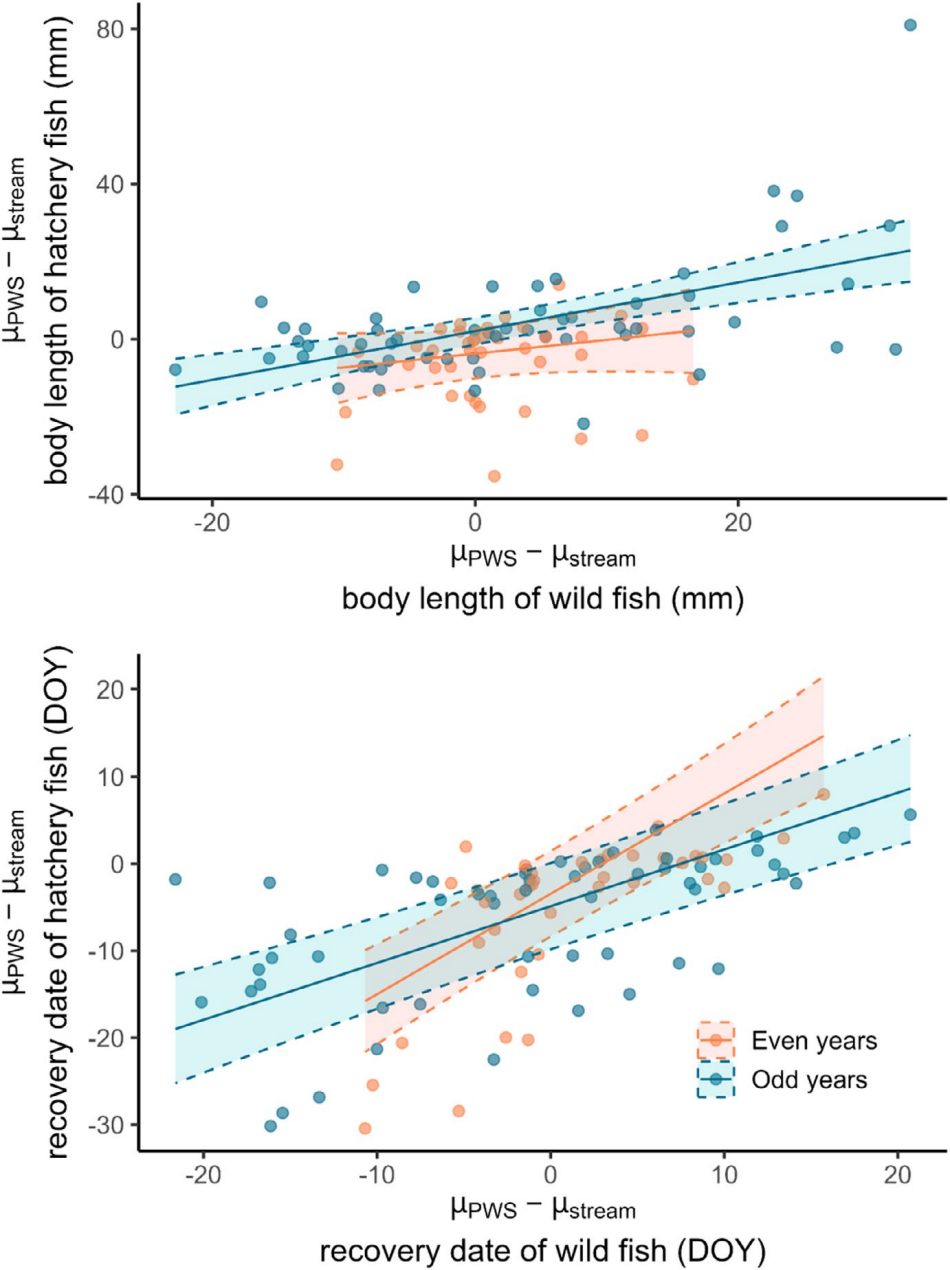
Evidence of phenotypic sorting of stray adult hatchery‐origin pink salmon returning to spawn in natural streams in Prince William Sound, AK. Regressions of the effect of the difference between the mean phenotype of fish in a given stream (μ_stream_) and the mean phenotype of fish among all streams (μ_PWS_) for wild fish (*x*‐axes) and hatchery‐origin fish (*y*‐axes). Phenotypes of body length (top) and recovery date (bottom) were modeled separately, as were even (orange) and odd (blue) year lineages. Empirical estimates (points) and significant fitted values (lines) are depicted. Model‐fit lines are bounded by 95% confidence intervals.

Preliminary analyses suggested that the body length and return timing of hatchery‐origin fish may be associated with the phenotypes of the wild populations they stray into; thus, we modeled hatchery phenotypes as a function of wild phenotypes. The null model best described the mean body length of hatchery fish in even‐years, and thus did not include the mean body length of wild fish (AICc = 291.2, *w*AICc = 0.70, *R* ^2^ = 0.15; Table S2), although the null model was only slightly better fit than a model containing an effect of mean body length of wild fish (ΔAICc = 1.66; *β*_Wild Length_ = 0.36, 95% CI [−0.20–0.92]; *R* ^2^ = 0.21). In odd‐years, hatchery fish length was best explained by wild fish length (*β*_Wild Length_ = 0.63, 95% CI [0.38–0.87]; AICc = 459.8, *w*AICc = 1.0, *R* ^2^ = 0.31). From these results, we concluded that larger hatchery strays returned to streams with wild populations that had larger body sizes (Figure 4), although this result was better supported in odd‐year populations.

The mean return timing of hatchery fish was best explained by the return timing of wild fish in even‐years (*β*_Wild DOY_ = 1.15, 95% CI [0.86–1.45]; AICc = 291.2, *w*AICc = 0.70, *R* ^2^ = 0.70) and in odd‐years (*β*_Wild DOY_ = 0.65, 95% CI [0.38–0.87]; AICc = 291.2, *w*AICc = 0.70, *R* ^2^ = 0.66). From these results, we concluded that straying was assortative (i.e., not random) with regards to timing: Earlier returning hatchery strays returned to earlier returning wild populations (Figure 4).

## 4 Discussion

We analyzed an extensive dataset of ca. 218,000 individual pink salmon sampled from 30 wild spawning populations in Prince William Sound, Alaska, over 8 years. Our findings highlighted that hatchery pink salmon returning to PWS between 2013 and 2020 were distinct from wild fish in body length, although the direction of this difference varied with lineage (even‐ or odd‐years). Additionally, across both lineages, straying hatchery salmon consistently entered spawning streams later than their wild counterparts, likely attributable in part to the original selection of relatively late spawning broodstock (Habicht, Simpson, and Seeb 2000). Hatchery strays also exhibited consistently reduced phenotypic variation in both body size and run timing than wild counterparts. Moreover, our findings revealed evidence consistent with phenotypic sorting: Hatchery‐origin fish predominantly strayed into populations where the wild‐origin fish had similar body sizes and return timings to their own. We did not examine the mechanisms underpinning this phenotypic sorting pattern; however, we discuss below how a suite of behavioral, genetic, and ecological factors may interact to generate observed patterns, which should be investigated by future studies. This work builds on analyses by Shedd et al. (2022) by providing context for how specific traits might differ by origin and how this might lead to differences in reproductive fitness addressed in their study. More broadly, our findings have important implications for how hatchery fish may or may not impact the genetic diversity, adaptive capacity, and productivity of wild salmon metapopulations.

Differences in body size, such as we detected between hatchery and wild pink salmon, form the basis for many nuanced interactions between genetic and environmental factors in salmonids. Body size is strongly controlled by both environmental and underlying genetic variation (Smoker et al. 1994; Carlson and Seamons 2008), which allows for allometric relationships with a number of traits such as freshwater and marine survival, fecundity, egg size, and competitive dominance, all of which are connected to lifetime reproductive success in salmonids (Fleming and Petersson 2001; Dickerson, Quinn, and Willson 2002; Garcia de Leaniz et al. 2007; Koch and Narum 2021). Yet, environmental factors can alter these relationships; for example, in shallow spawning streams, larger body sizes can hamper mobility (Peterson, Hilborn, and Hauser 2014) or increase predation risk (Lin et al. 2016), leading to selection against increased body size. In natural pink salmon populations, males are generally larger than females (Heard 1991). We found that hatchery females exhibited the greatest body length in even‐years, suggesting sex‐specific hatchery effects on body size. Given that body size is associated with high heritability values and influences sexual competition and reproductive success (Funk et al. 2005), our results highlight the possibility for hatcheries to affect wild population dynamics within generations through effects on competition and among generations through genetic introgression. Our finding that hatchery fish were larger than wild fish in even return years but smaller in odd return years could indicate different responses of hatchery and wild fish to at‐sea competition. This biennial pattern in return abundance appears to be maintained in both wild and hatchery pink salmon in PWS. Survival of hatchery pink salmon tends to be higher in odd brood year cohorts (fry released in even‐years) when wild fry are more abundant (Heard and Wertheimer 2012). Heard and Wertheimer (2012) cited differences in diets of juveniles as a potential driver, but this pattern could also result from predator swamping. This may lead to the potential for a greater effect of competition at sea, among both wild and hatchery‐origin fish, for the odd‐year line. Smaller hatchery fish emerging from higher competition environments may indicate that wild fish have a competitive advantage over hatchery fish and this hypothesis should be explored in future studies. Furthermore, differences in body size between hatchery and wild fish may partially explain reduced relative reproductive success (RRS) of hatchery‐origin fish that has been reported in this system (Shedd et al. 2022). Our finding of reduced variation in body size in hatchery‐origin fish is consistent with a number of previous studies (Quinn et al. 2002; Chittenden et al. 2010; McPhee et al. 2024), despite efforts by PWS hatchery managers to maximize genetic diversity by using many, randomly selected broodstock. These findings warrant concern, as many generations of hatchery‐wild interbreeding could reduce variation in body size in the wild, which may subsequently lead to decreased population resilience through reduced portfolio effects (Schindler, Armstrong, and Reed 2015).

In addition to divergence in body size, we also consistently observed hatchery‐origin individuals returning later than wild fish (see also Knudsen et al. 2021) which may offer some insight to possible mechanisms driving domestication selection in hatcheries or the reduced RRS of hatchery fish (Shedd et al. 2022). Although we assume that carcass recovery date is representative of return timing, we do not expect that any violation of this assumption would differ by fish origin. Notably, original broodstock selection for hatcheries in PWS in the 1960's favored later‐returning fish spawning above the high‐tide line, because of perceived compatibility with freshwater hatchery rearing conditions and a desire to temporally segregate runs to aid cost recovery and common property fisheries targeting hatchery returns (Habicht, Simpson, and Seeb 2000). Like body size, return timing and other phenological traits have been found to have moderate to high heritability values in salmon (Carlson and Seamons 2008), and are controlled by few loci of large effect in some cases (Waples et al. 2022). In addition, broodstock for PWS hatcheries are taken from adults returning to hatcheries, not from wild streams (i.e., a segregated hatchery system). Thus, one likely explanation for our finding of delayed hatchery timing is a genetic legacy of the original broodstock selection of later returning fish. The effect of this timing mismatch on wild population recruitment and resilience remains unclear, but it is likely that hatchery straying with consistently divergent run timing from wild populations could affect the genetic and phenotypic diversity of wild populations (May et al. 2024). Consistent straying and annual interbreeding of hatchery fish with natural populations may induce a phenological shift in wild populations (Tillotson et al. 2019). Given that timing is strongly linked to fitness, such a phenological shift could reduce the productivity of natural populations. More specifically, early and late spawning pink salmon possess distinct embryonic development rates, with the former having a slower rate and the latter a faster one; this counter‐gradient variation aligns fry emigration timing with the most favorable juvenile growth conditions (Echave et al. 2017). Furthermore, greater genetic variation in emergence timing exists for early run individuals and may ensure population persistence through spawning temperatures that are more variable than for the later run (Hebert et al. 1998). Thus, selection regimes for emergence timing, incubation temperatures, juvenile growth, and migration timing likely differ between the hatchery and wild population segments. In addition to evolutionary effects, later returning hatchery‐origin strays may have within‐cohort impacts such as physical displacement of the eggs of earlier‐returning wild fish (i.e., redd superimposition) or reduced dissolved oxygen levels in the stream if high population densities coincide with elevated temperatures (Sergeant et al. 2017). Further investigation is warranted into the mechanisms underlying these hatchery‐wild differences and their ultimate consequences for wild population recruitment and resilience.

Phenotypic sorting of strays, where hatchery‐origin salmon strayed into populations with natural‐origin fish of similar body sizes and return timings to their own, emerged as a salient finding in our study. We characterize this phenotypic sorting pattern as a specific type of phenotype‐dependent dispersal syndrome where phenotypes covary among immigrants and local individuals (Ronce and Clobert 2012). This pattern could stem from at least three, non‐mutually exclusive mechanisms: Matching habitat choice, hatchery effects, or collective navigation. Matching habitat choice suggests that individuals actively select habitats or populations where their phenotype, in this case body size and return timing, provides the highest fitness (Quinn, Kinnison, and Unwin 2001; Edelaar, Siepielski, and Clobert 2008; Camacho and Hendry 2020), aligning well with the observations that hatchery fish stray to areas with similar‐sized wild fish. Hatchery practices may also affect the spatial distribution of strays, particularly because hatchery strays tend to stray to natural populations near their release sites. The segregated hatcheries in PWS use returning hatchery‐origin individuals as broodstock each generation. If the natural phenotypic variation documented in this study varies spatially within PWS, then broodstock selection or juvenile release practices may also contribute to the phenotypic sorting pattern. In a supplemental analysis, we found evidence for phenotypic variation among some hatcheries (Figure S3), which may support this mechanism. In addition to spatial variation, another hatchery effect could come from environmental variation in hatchery conditions that contributes to variation in imprinting and ultimately results in plastic homing responses to environmental cues for returning adults. A third mechanism, collective movement, suggests that fish might move as coordinated groups (Berdahl et al. 2016; Westley et al. 2018), leading to areas where their collective attributes, like body size or timing, may be similar (also related to “contingent migration” and the “stock concept” Secor 1999). Importantly, matching habitat choice, hatchery effects, and collective navigation are not mutually exclusive and could each contribute to this pattern (Bolnick et al. 2009). Although discerning the mechanisms underlying observed phenotypic sorting was outside the scope of this study, an interesting avenue for future research will be to partition the variance in spatial sorting according to each of the mechanisms proposed here. Such studies might further investigate phenotypic or genetic differences among specific hatcheries, spatial or temporal correlations among hatcheries and wild populations, spatial or temporal variation in rates of natural straying compared to hatchery strays, or variation in pedigree‐based estimates of lifetime reproductive success within and among streams.

Another important insight provided by this analysis was that there exists substantial variation in both body size and timing among pink salmon populations in PWS. Owing to their large population sizes and assumed high straying rates, pink salmon are often thought of as genetically and phenotypically homogenous. Challenging this notion, the large‐scale nature of our study uncovered significant variation in average body and spawn timing between populations. Such variation is likely adaptive and, while not as striking as the life‐history variation observed in other salmon species, may equally contribute to population productivity and resilience. One underlying assumption in our study is that natural‐origin fish were assumed to return to natal streams, whereas some likely stray, introducing a source of potential bias. While natural straying rates within PWS—and immigration rates from outside regions—remain uncertain, preliminary findings in a previous study suggest approximately 3% of natural‐origin spawners were strays among four nearby streams (May et al. 2023). Donor rates of straying (i.e., the percentage of individuals originating in a given stream that were shown to return to neighboring streams within a 35 km radius of the natal watershed) was estimated to be 5.3% for wild pink salmon in a study in southeast AK (Wertheimer et al. 2000). Stray rates among more geographically distant populations are likely even lower. Thus, it is unlikely that natural‐origin strays had a major influence on our results. However, the observed phenotypic variation among PWS populations suggests that natural‐origin straying may decrease phenotypic differentiation among populations by introducing phenotypes from other areas. Consequently, this process could increase the likelihood of Type II errors in detecting phenotypic sorting in our study, potentially underestimating the true magnitude of phenotypic sorting. While we consider this effect unlikely to substantially alter our main conclusions, it presents an interesting area for future study, especially regarding how natural‐origin straying might obscure or amplify phenotypic sorting.

An intriguing consequence of phenotypic sorting of dispersers is that it may reduce the magnitude of phenotypic mismatch between hatchery and wild fish and thus potentially affect the ways in which hatchery fish influence wild populations. Wild salmon populations are typically adapted to their local environments (Fraser et al. 2011). As such, phenotypic sorting may reduce the degree of maladaptation of hatchery‐origin individuals to local environments, compared to random straying of hatchery individuals. Phenotypic sorting may also reinforce local adaptation or reduce the degree of adaptive erosion compared to random straying (Edelaar, Siepielski, and Clobert 2008; Camacho and Hendry 2020). While the relative reproductive success (RRS) of hatchery fish is reduced compared to wild individuals in PWS (Shedd et al. 2022), phenotypic sorting may serve to increase RRS compared to what it would be under random dispersal, because of reduced phenotypic mismatch (May et al. 2024). Thus, we hypothesize that phenotypic sorting may increase ultimate metapopulation productivity but also increase the rate of introgression of hatchery origin alleles into wild populations. Further investigation into the consequences of phenotypic sorting on the productivity and adaptive capacity of wild populations is warranted.

In conclusion, our large‐scale, phenotypic analysis of pink salmon offers novel insights into the nuanced morphological and behavioral differences spanning PWS hatchery and wild fish. Results challenge the traditional view of pink salmon as phenotypically homogenous and underscore the intricate phenotypic diversity within the species. Recent years have seen growing concern over the possible impacts of large‐scale production hatcheries on wild salmon populations (Naish et al. 2008; McMillan et al. 2023), highlighting potential risks like decreased recruitment (Amoroso, Tillotson, and Hilborn 2017; Cunningham, Westley, and Adkison 2018; Ohlberger et al. 2022; Jaeger and Scheuerell 2023), reduced genetic or phenotypic diversity (Jasper et al. 2013; Perrier et al. 2013; Christie et al. 2016; Willoughby and Christie 2019), and potential ecosystem destabilization (Tillotson et al. 2019; Terui et al. 2023). Our findings reaffirm the validity of some of these concerns, underscoring the need for further research into drivers of observed differences and outcomes for wild salmon populations. Yet, despite many generations of hatchery introgression, significant variation still exists among wild populations, evidenced by our phenotypic sorting findings. As fisheries become increasingly reliant on hatchery production, understanding ecological and genetic ramifications for wild populations remains paramount to reform management strategies for these economically and culturally significant fisheries. The intertwined dynamics of hatchery practices, wild salmon population conservation, and fisheries management present considerable challenges that demand informed, evidence‐based strategies (Lichatowich 2001; Brannon et al. 2004; Rand et al. 2012; Gayeski et al. 2018). Our study contributes to this endeavor, emphasizing the importance of continued large‐scale monitoring, adaptive management, and conservation efforts to sustain the vitality and diversity of salmon populations for generations to come.

## Author Contributions

**Julia McMahon:** conceptualization (equal), data curation (lead), formal analysis (equal), funding acquisition (supporting), investigation (lead), methodology (equal), writing – original draft (supporting). **Samuel A. May:** conceptualization (equal), formal analysis (equal), funding acquisition (supporting), investigation (equal), methodology (equal), visualization (equal), writing – original draft (lead). **Peter S. Rand:** conceptualization (supporting), data curation (equal), funding acquisition (supporting), investigation (supporting), methodology (supporting), resources (equal), writing – review and editing (supporting). **Kristen B. Gorman:** conceptualization (supporting), data curation (equal), funding acquisition (supporting), investigation (supporting), methodology (supporting), resources (equal), writing – review and editing (supporting). **Megan V. McPhee:** conceptualization (supporting), investigation (supporting), methodology (supporting), supervision (supporting), writing – review and editing (supporting). **Peter A. H. Westley:** conceptualization (equal), data curation (supporting), formal analysis (supporting), funding acquisition (lead), investigation (supporting), methodology (supporting), project administration (lead), resources (supporting), supervision (lead), writing – review and editing (equal).

## Conflicts of Interest

The authors declare no conflicts of interest.

## Supporting information


Data S1.

## Data Availability

This submission uses publicly accessible novel code and data available at the following link: https://github.com/SMay1/Pink_Salmon_Hatchery_Wild_Differences_PWS. These data will be permanently archived on Zenodo upon publication.
