# Supplementary material for: Phenotypic Sorting of Pink Salmon Hatchery Strays May Alleviate Adverse Impacts of Reduced Variation in Fitness‐Associated Traits
Source: Ecol Evol. 2025 Jan 8;15(1):e70781. doi: 10.1002/ece3.70781 (PMC11711052; doi:10.1002/ece3.70781)
Supplement: Supplementary file 1 — Data S1. [file ECE3-15-e70781-s001.zip › Phenotypic_Sorting_Pink_Salmon_Supplement.pdf]

Supplementary Materials for

Phenotypic sorting of pink salmon hatchery strays may alleviate adverse impacts of reduced variation in fitness-associated traits

Julia McMahon & Samuel A. May *et al.*

*Corresponding author. pwestley@alaska.edu

This PDF file includes:

Figs. S1 to S3
Tables S1 to S2

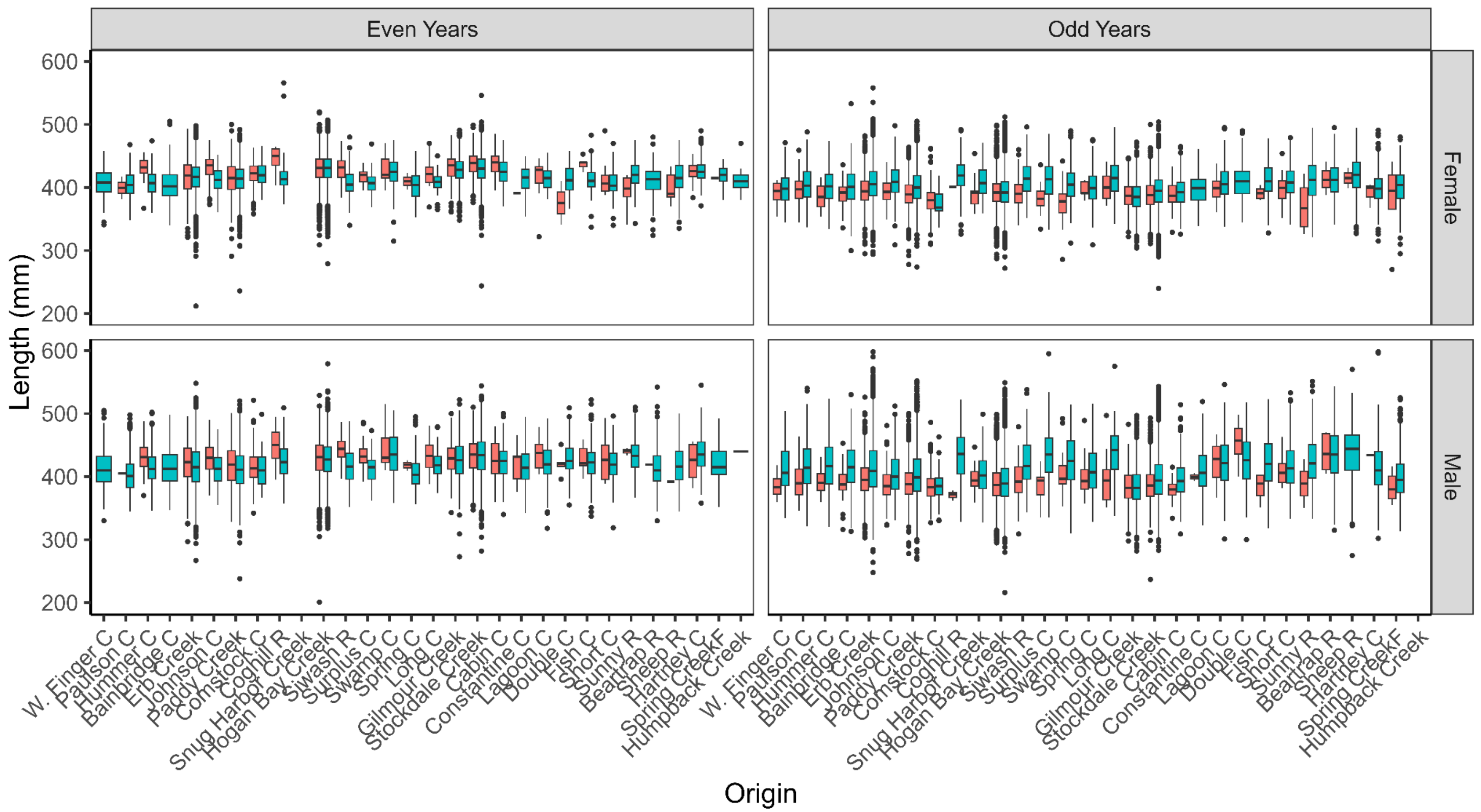

Fig. S1.

Box plots (medians, quantiles, and outlier points) comparing body size (mm) of hatchery-origin (red) to wild-origin (blue) pink salmon for each AHRP sample stream (x-axes), sex (male or female), and lineage (even or odd years). Sample streams are ordered from west to east.

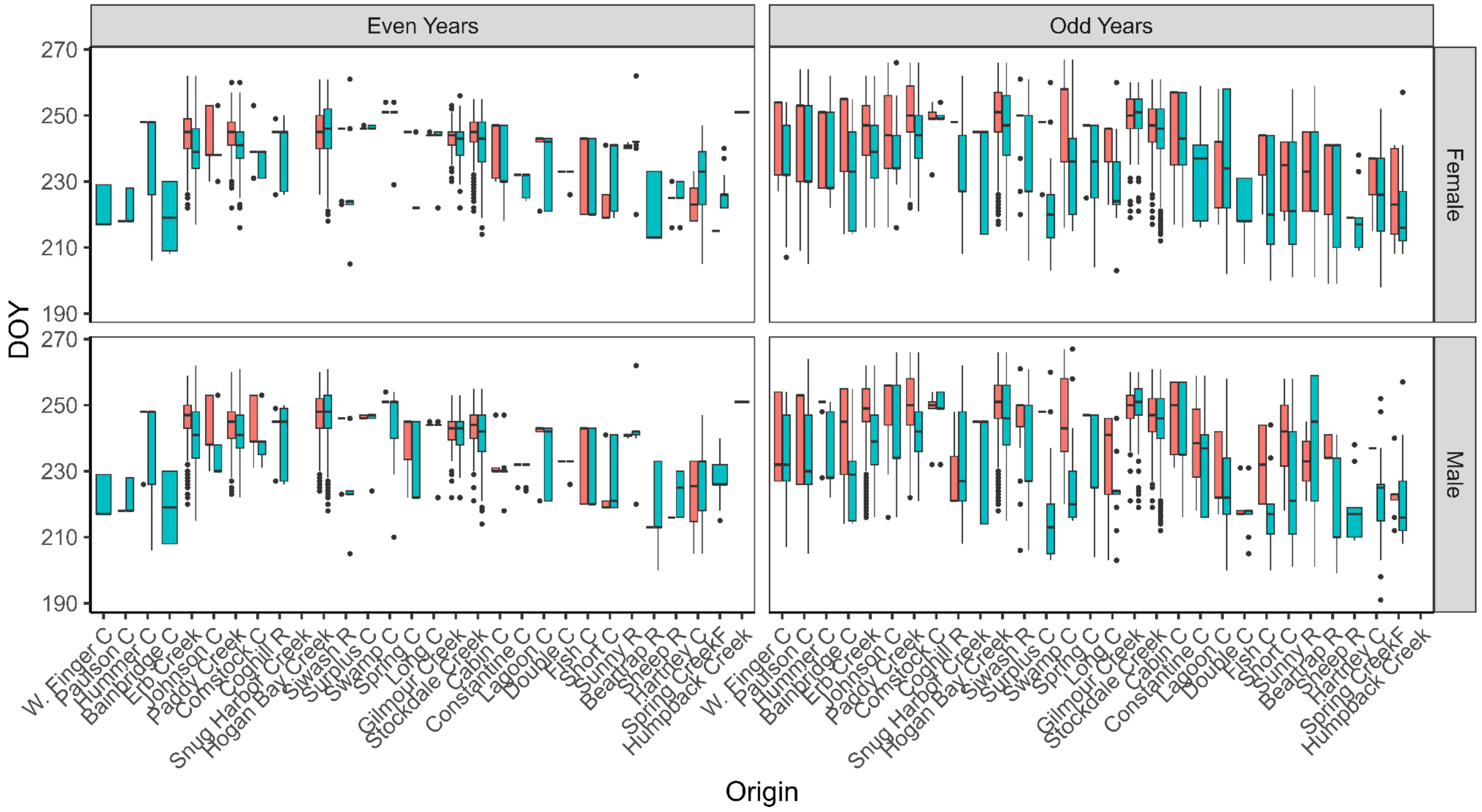

Fig. S2.

Box plots (medians, quantiles, and outlier points) comparing day of year sampled (DOY) of hatchery-origin (red) to wild-origin (blue) pink salmon for each AHRP sample stream (x-axes), sex (male or female), and lineage (even or odd years). Sample streams are ordered from west to east.

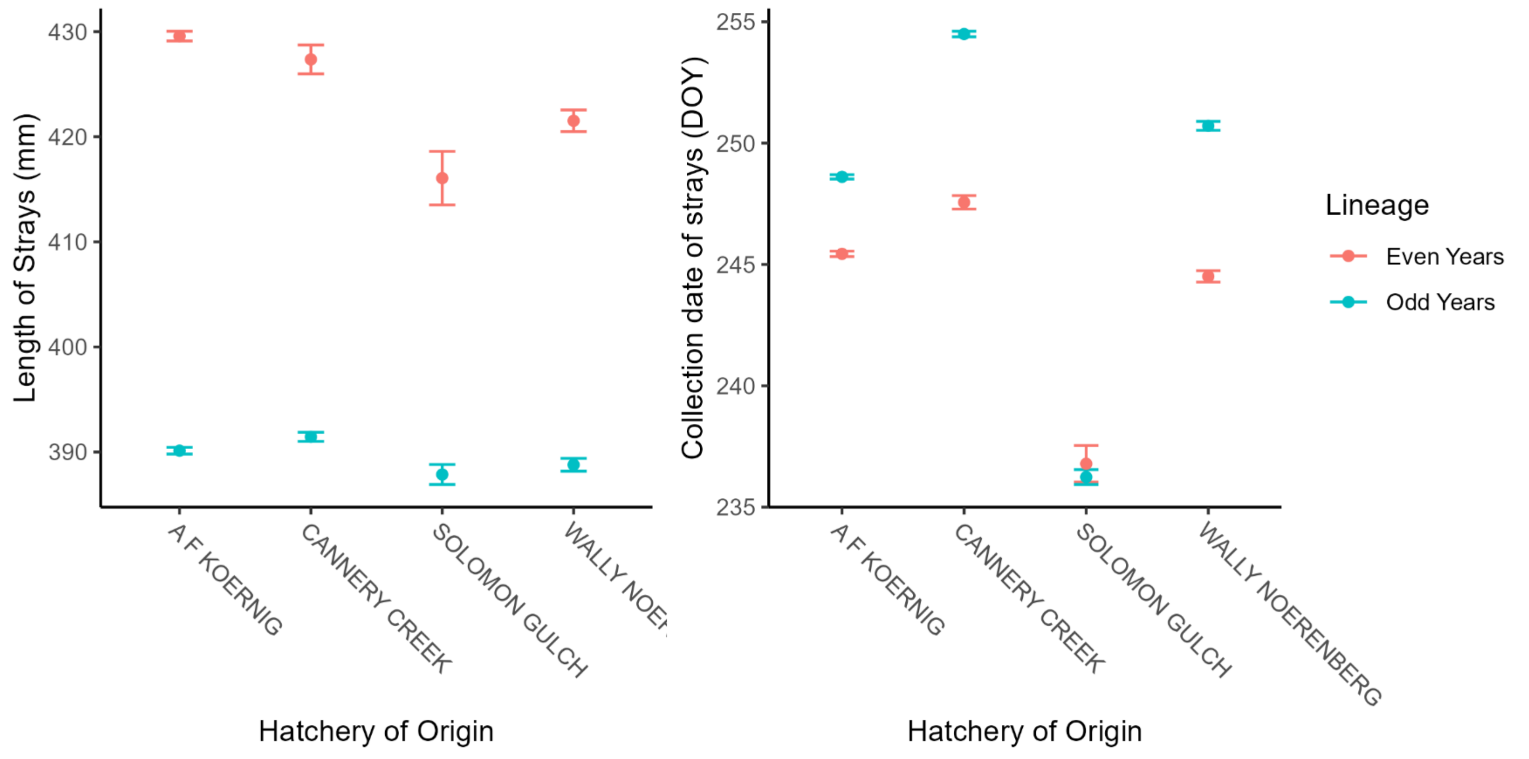

Fig. S3.

Phenotypic differences among adult, hatchery-origin pink salmon sampled in natural streams in PWS, AK. Mean phenotypic values (y-axes) from all strays collected from all streams are provided for body length (mm; left) and collection date (DOY; right). Hatchery of origin (x-axis) was determined using hatchery-specific thermally marked otoliths. Values are colored by lineage, and error bars represent 95% confidence intervals.

Table S1.

Mean mid-eye to hypural-plate body length (mm), standard deviation, and the sample size of otoliths read of hatchery (H) and wild (W) origin sampled from 2013 to 2018. “--” indicates that no hatchery or wild samples were identified at that creek, year, and origin category. Sample location followed by the map number from Figure 1 and the ADF&G anadromous waters catalog number.

Location	Origin	2013	2014	2015	2016	2017	2018	2019	2020
Bainbridge C	H	395, 25, 25	--	388, 22, 65	--	--	--	--	--
(3; 226-20-16300)	W	404, 30, 219	410, 28, 476	414, 36, 508	--	--	--	--	--
Beartrap R	H	437, 33, 3	419, --, 1	424, 31, 5	--	--	--	--	--
(16; 221-30-1048)	W	432, 42, 367	413, 30, 459	424, 34, 448	--	--	--	--	--
Cabin C	H	392, 20, 11	434, 25, 56	384, 22, 52	--	--	--	--	--
(9; 227-20-17464)	W	400, 27, 221	423, 25, 298	393, 28, 489	--	--	--	--	--
Coghill R	H	378, 17, 4	448, 27, 24		--	--	--	--	--
(25; 223-30-13220)	W	416, 33, 138	421, 28, 222	429, 34, 461	--	--	--	--	--
Comstock C	H	396, 26, 107	417, 24, 441	378, 22, 348	--	--	--	--	--
(29; 225-20-15040)	W	394, 33, 11	415, 25, 67	379, 22, 76	--	--	--	--	--
Constantine C	H	--	416, 33, 7	400, 9, 2	--	--	--	--	--
(11; 228-60-18150)	W	405, 30, 294	415, 25, 269	406, 32, 528	--	--	--	--	--
Double C	H	400, --, 1	408, 34, 7	467, 26, 4	--	--	--	--	--

(12; 228-40-18310)	W	414, 36, 574	423, 26, 130	435, 33, 366	--	--	--	--	--
Erb C	H	394, 24, 43	430, 23, 402	387, 23, 1967	444, 22, 354	403, 26, 2267	422, 24, 271	--	400, 25, 733
(2; 226-20-16040)	W	401, 26, 385	406, 27, 1087	403, 31, 9315	434, 24, 3528	418, 38, 9901	416, 28, 7574	--	406, 27, 6004
Fish C	H	--	431, 18, 16	388, 16, 11	--	--	--	--	--
(19; 221-40-10890)	W	420, 35, 312	419, 27, 414	418, 36, 577	--	--	--	--	--
Gilmour C	H	--	430, 22, 317	389, 22, 665	434, 22, 137	400, 20, 78	424, 29, 124	381, 24, 856	--
(8; 227-20-17480)	W	--	423, 23, 246	387, 26, 2108	434, 24, 1018	398, 27, 261	421, 28, 1045	382, 26, 2702	--
Hartney C	H	402, 3, 3	423, 24, 15	395, 25, 5	--	--	--	--	--
(13; 221-10-10020)	W	400, 25, 436	432, 27, 203	414, 39, 526	--	--	--	--	--
Hogan Bay C	H	405, 22, 426	440, 23, 2253	385, 23, 4806	441, 22, 2445	396, 23, 10927	420, 25, 5313	387, 25, 13212	--
(5; 226-30-16810)	W	400, 26, 263	426, 27, 233	395, 31, 4128	437, 22, 9695	398, 32, 6204	417, 26, 7812	386, 28, 4495	--
Hummer C	H	414, 16, 2	432, 21, 81	387, 19, 36	--	--	--	--	--
(26; 224-10-14240)	W	403, 30, 128	411, 25, 308	413, 36, 383	--	--	--	--	--
Humpback C (221-10-10110)	W	--	411, 16, 46	--	--	--	--	--	--
Johnson C	H	393, 26, 43	431, 22, 330	391, 24, 157	--	--	--	--	--
(4; 226-40-16269)	W	399, 24, 95	412, 23, 155	408, 32, 448	--	--	--	--	--
Lagoon C	H	391, 17, 5	428, 32, 23	415, 31, 16	--	--	--	--	--

(20; 221-40-10990)	W	408, 32, 468	420, 26, 390	425, 36, 545	--	--	--	--	--
Long C	H	401, 8, 3	431, 24, 71	400, 31, 30	--	--	--	--	--
(21; 222-10-12140)	W	413, 32, 89	415, 21, 88	430, 35, 400	--	--	--	--	--
Paddy C	H	410, 26, 8	430, 23, 555	386, 22, 2707	444, 20, 157	400, 27, 1234	413, 27, 104	--	401, 24, 841
(3; 226-20-16010)	W	391, 22, 70	415, 27, 339	396, 29, 5577	438, 22, 700	413, 36, 4397	416, 28, 1991	--	407, 26, 4934
Paulson C	H	393, 28, 18	401, 18, 3	397, 23, 63	--	--	--	--	--
(27; 224-10-14550)	W	399, 28, 260	405, 28, 455	418, 35, 473	--	--	--	--	--
Sheep R	H	--	398, 24, 6	415, 23, 2	--	--	--	--	--
(15; 221-20-10360)	W	427, 38, 695	417, 30, 417	433, 35, 554	--	--	--	--	--
Short C	H	402, 31, 7	419, 29, 30	403, 25, 14	--	--	--	--	--
(18; 221-40-10880)	W	411, 31, 1093	415, 26, 306	417, 33, 436	--	--	--	--	--
Siwash R	H	414, 24, 16	437, 20, 151	390, 25, 118	--	--	--	--	--
(24; 222-20-12640)	W	409, 29, 138	414, 28, 302	421, 33, 463	--	--	--	--	--
Snug Harbor C	H	--	--	393, 24, 25	--	--	--	--	--
(6; 226-30-16820)	W	--	--	406, 30, 265	--	--	--	--	--
Spring C	H	--	415, 12, 5	401, 33, 14	--	--	--	--	--
(22; 222-10-12170)	W	391, 28, 67	405, 24, 294	408, 32, 570	--	--	--	--	--
Spring C	H	387, 34, 26	415, --, 1	372, 9, 2	--	--	--	--	--

(14; 221-20-10200)	W	400, 31, 1224	419, 22, 93	401, 32, 376	--	--	--	--	--
Stockdale C	H	395, 21, 157	434, 23, 895	385, 24, 1656	442, 22, 583	400, 25, 1036	426, 27, 219	381, 25, 1290	--
(10; 227-20-17520)	W	392, 22, 851	420, 27, 395	388, 29, 6655	439, 25, 7208	411, 32, 9617	420, 27, 4372	384, 27, 6955	--
Sunny R	H	--	409, 33, 9	377, 43, 6	--	--	--	--	--
(17; 221-40-10875)	W	419, 35, 260	428, 27, 295	419, 36, 378	--	--	--	--	--
Surplus C	H	--	427, 21, 51	383, 22, 34	--	--	--	--	--
(23; 222-20-12338)	W	419, 35, 371	410, 21, 119	433, 35, 447	--	--	--	--	--
Swamp C	H	397, 26, 12	436, 30, 21	384, 32, 55	--	--	--	--	--
(7; 227-20-17390)	W	397, 27, 259	428, 27, 187	429, 36, 526	--	--	--	--	--
West Finger C	H	387, 15, 7	--	389, 22, 12	--	--	--	--	--
(28; 224-40-14850)	W	397, 24, 220	411, 26, 448	412, 36, 389	--	--	--	--	--

Table S2.

Results of stepwise generalized linear model selection procedures. Models were evaluated with the second-order Akaike Information Criterion (AICc). All models were compared to the best-fit model by comparing AICc values (ΔAICc). The weighted AIC score (wAICc) is the proportion of the total predictive power provided by the full set of models. Log-likelihood (LL) accounts for the number of model parameters (K; degrees of freedom). The null model refers to an intercept-only model with no covariates. Response variables are provided for different suites of sub-models, alongside the data subset used in that mode (i.e., even or odd years).

**Response**	**Model**	**K**	**LL**	**AICc**	**ΔAICc**	***w*AICc**
Even Length	DOY + Origin + Sex + (1 | Stream) + (1 | Year)	7	-380581	761176.1	0	0.42
	DOY + Origin + Sex + (1 | Stream) + (1 | Year) + Origin:Sex	8	-380581	761177	0.9	0.27
	DOY + Origin + Sex + (1 | Stream) + (1 | Year) + DOY:Sex	8	-380581	761178.1	1.92	0.16
	DOY + Origin + Sex + (1 | Stream) + (1 | Year) + DOY:Sex + Origin:Sex	9	-380580	761178.5	2.36	0.13
	DOY + Origin + (1 | Stream) + (1 | Year)	6	-380586	761183.2	7.08	0.01
	Origin + Sex + (1 | Stream) + (1 | Year)	6	-380586	761183.5	7.37	0.01
	Origin + Sex + (1 | Stream) + (1 | Year) + Origin:Sex	7	-380585	761184.3	8.14	0.01
	Origin + (1 | Stream) + (1 | Year)	5	-380591	761191.7	15.58	0
	Sex + (1 | Stream) + (1 | Year)	5	-380774	761558.5	382.39	0
	DOY + Sex + (1 | Stream) + (1 | Year)	6	-380775	761561	384.86	0
	DOY + Sex + (1 | Stream) + (1 | Year) + DOY:Sex	7	-380774	761562.1	385.91	0
	NULL	4	-380780	761567.5	391.37	0
	DOY + (1 | Stream) + (1 | Year)	5	-380780	761569.5	393.38	0
Odd Length	DOY + Origin + Sex + (1 | Stream) + (1 | Year) + DOY:Sex + Origin:Sex	9	-655065	1310148	0	1

	DOY + Origin + Sex + (1 | Stream) + (1 | Year) + DOY:Sex	8	-655106	1310228	79.85	0
	DOY + Sex + (1 | Stream) + (1 | Year) + DOY:Sex	7	-655244	1310502	353.86	0
	DOY + Origin + Sex + (1 | Stream) + (1 | Year) + Origin:Sex	8	-655497	1311010	862.14	0
	DOY + Origin + Sex + (1 | Stream) + (1 | Year)	7	-655690	1311394	1246.16	0
	DOY + Sex + (1 | Stream) + (1 | Year)	6	-655832	1311676	1527.9	0
	DOY + Origin + (1 | Stream) + (1 | Year)	6	-655833	1311678	1529.6	0
	DOY + (1 | Stream) + (1 | Year)	5	-655978	1311966	1817.97	0
	Origin + Sex + (1 | Stream) + (1 | Year) + Origin:Sex	7	-659435	1318885	8736.45	0
	Origin + Sex + (1 | Stream) + (1 | Year)	6	-659669	1319350	9201.37	0
	Origin + (1 | Stream) + (1 | Year)	5	-659827	1319664	9515.62	0
	Sex + (1 | Stream) + (1 | Year)	5	-660219	1320447	10298.79	0
	NULL	4	-79837	1320778	10629.21	0
Even Timing	Origin + Sex + (1 | Stream) + (1 | Year) + Origin:Sex	7	-79839	159688	0	0.96
	Length + Origin + Sex + (1 | Stream) + (1 | Year) + Origin:Sex	8	-79846	159694.2	6.16	0.04
	Length + Origin + Sex + (1 | Stream) + (1 | Year) + Length:Sex + Origin:Sex	9	-79850	159709	21.01	0
	Origin + Sex + (1 | Stream) + (1 | Year)	6	-79852	159712.2	24.22	0
	Length + Origin + Sex + (1 | Stream) + (1 | Year)	7	-79858	159718.2	30.24	0
	Length + Origin + Sex + (1 | Stream) + (1 | Year) + Length:Sex	8	-79943	159731.3	43.34	0

	Origin + (1 | Stream) + (1 | Year)	5	-79945	159896.6	208.64	0
	Length + Origin + (1 | Stream) + (1 | Year)	6	-80314	159901.5	213.47	0
	Sex + (1 | Stream) + (1 | Year)	5	-80322	160638.8	950.83	0
	Length + Sex + (1 | Stream) + (1 | Year)	6	-80327	160655.4	967.44	0
	Length + Sex + (1 | Stream) + (1 | Year) + Length:Sex	7	-80404	160668.2	980.15	0
	NULL	4	-80411	160816	1127.98	0
	Length + (1 | Stream) + (1 | Year)	5	-79837	160832.1	1144.06	0
Odd Timing	Length + Origin + Sex + (1 | Stream) + (1 | Year) + Length:Sex + Origin:Sex	9	-173163	346343.5	0	1
	Length + Origin + Sex + (1 | Stream) + (1 | Year) + Origin:Sex	8	-173177	346369.1	25.54	0
	Length + Origin + Sex + (1 | Stream) + (1 | Year) + Length:Sex	8	-173181	346378.8	35.23	0
	Length + Origin + (1 | Stream) + (1 | Year)	6	-173186	346383.4	39.84	0
	Length + Origin + Sex + (1 | Stream) + (1 | Year)	7	-173189	346391.9	48.32	0
	Length + Sex + (1 | Stream) + (1 | Year) + Length:Sex	7	-175157	350327.3	3983.73	0
	Length + (1 | Stream) + (1 | Year)	5	-175165	350339.6	3996.06	0
	Length + Sex + (1 | Stream) + (1 | Year)	6	-175167	350345.5	4001.92	0
	Origin + Sex + (1 | Stream) + (1 | Year) + Origin:Sex	7	-177099	354212.4	7868.83	0
	Origin + Sex + (1 | Stream) + (1 | Year)	6	-177152	354315.9	7972.34	0
	Origin + (1 | Stream) + (1 | Year)	5	-177164	354338.1	7994.51	0

	Sex + (1 | Stream) + (1 | Year)	5	-179537	359083.6	12740.06	0
	NULL	4	-179555	359117.9	12774.4	0
Difference in Even Year Mean Hatchery Length (PWS - Stream)	NULL	3	-142	291.2	0	0.70
	Difference in Mean Wild Length (PWS - Stream)	4	-142	292.9	1.66	0.30
Difference in Odd-Year Mean Hatchery Length (PWS - Stream)	Difference in Mean Wild Length (PWS - Stream)	4	-225	459.8	0	1
	NULL	3	-234	474.6	14.86	0
Difference in Even-Year Mean Hatchery Return Day (PWS - Stream)	Mean Wild Return Day (PWS - Stream)	4	-120	251.0	0	1
	NULL	3	-136	280.1	29.16	0
Difference in Odd-Year Mean	Mean Wild Return Day (PWS - Stream)	4	-186	380.2	0	1

Hatchery Return Day (PWS - Stream)	NULL	3	-202	410.5	30.27	0
