# Supplementary material for: Phenotypic Sorting of Pink Salmon Hatchery Strays May Alleviate Adverse Impacts of Reduced Variation in Fitness‐Associated Traits
Source: Ecol Evol. 2025 Jan 8;15(1):e70781. doi: 10.1002/ece3.70781 (PMC11711052; doi:10.1002/ece3.70781)
Supplement: Supplementary file 1 — Data S1. [file ECE3-15-e70781-s001.zip › Supplementary Materials.docx]

Supplementary Materials for

**Phenotypic sorting of pink salmon hatchery strays may alleviate adverse impacts of reduced variation in fitness-associated traits**

Julia McMahon & Samuel A. May *et al.*

*Corresponding author. [pwestley@alaska.edu](mailto:pwestley@alaska.edu)

**This PDF file includes:**

Figs. S1 to S3

Tables S1 to S2


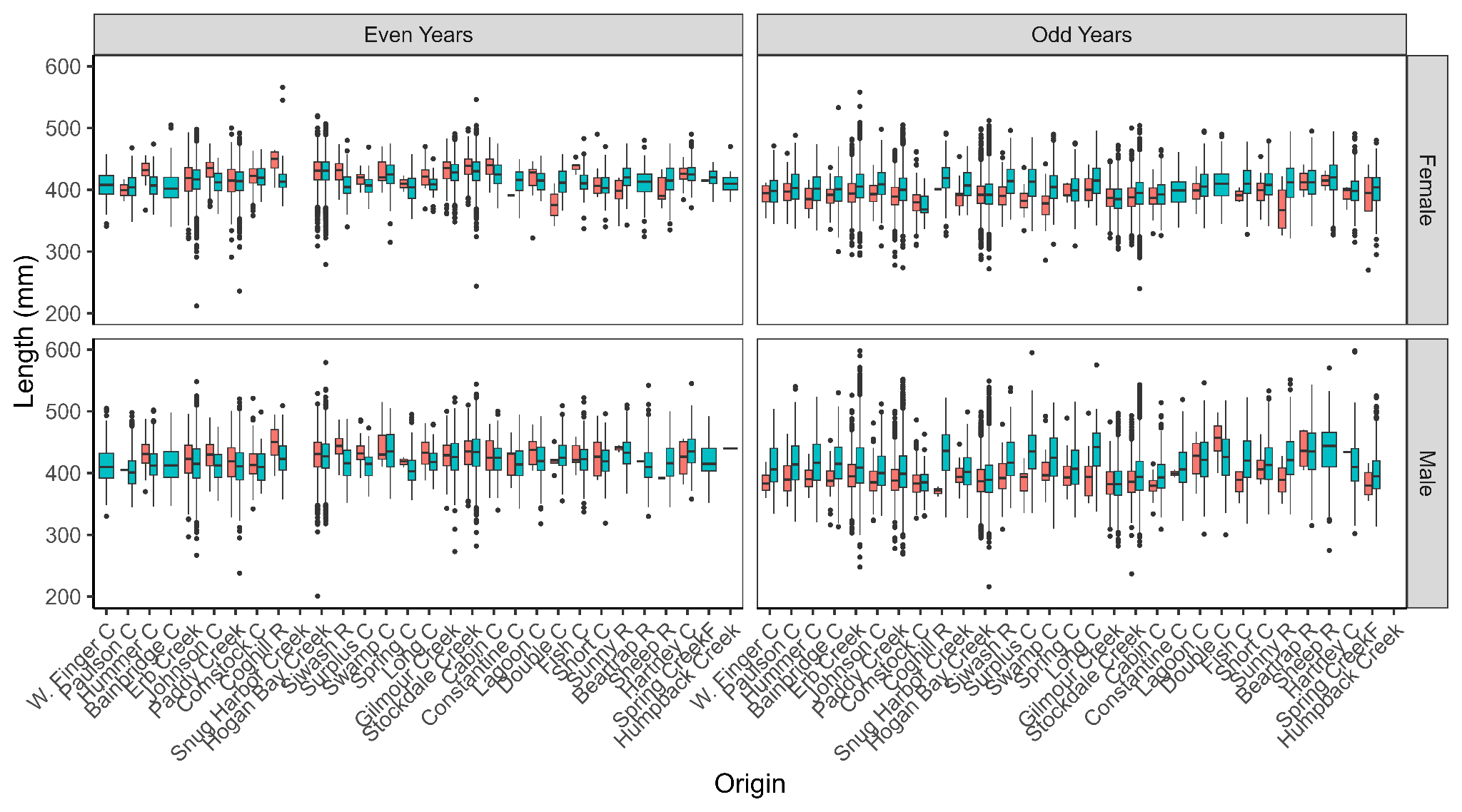


Fig. S1.

Box plots (medians, quantiles, and outlier points) comparing body size (mm) of hatchery-origin (red) to wild-origin (blue) pink salmon for each AHRP sample stream (x-axes), sex (male or female), and lineage (even or odd years). Sample streams are ordered from west to east.


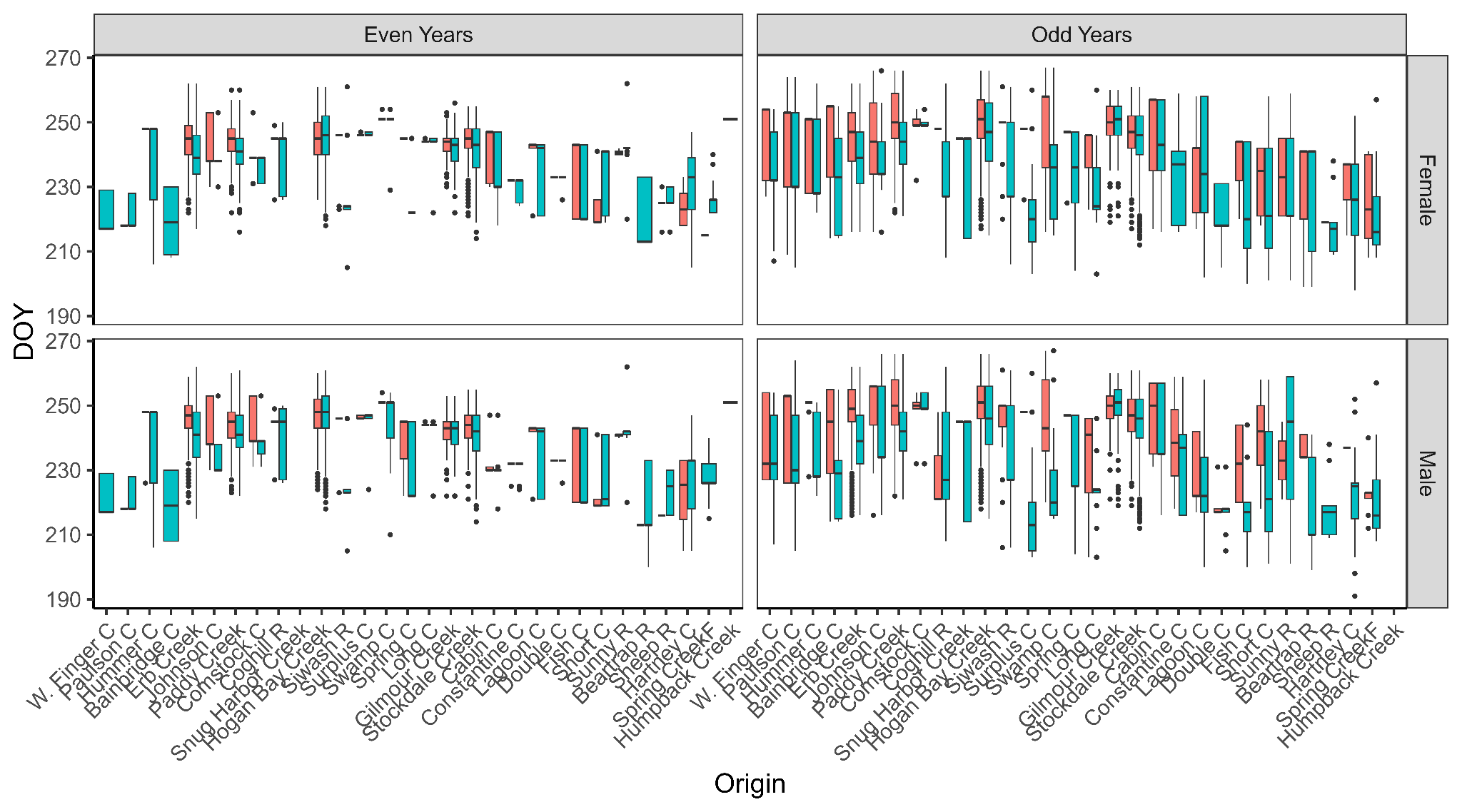


Fig. S2.

Box plots (medians, quantiles, and outlier points) comparing day of year sampled (DOY) of hatchery-origin (red) to wild-origin (blue) pink salmon for each AHRP sample stream (x-axes), sex (male or female), and lineage (even or odd years). Sample streams are ordered from west to east.


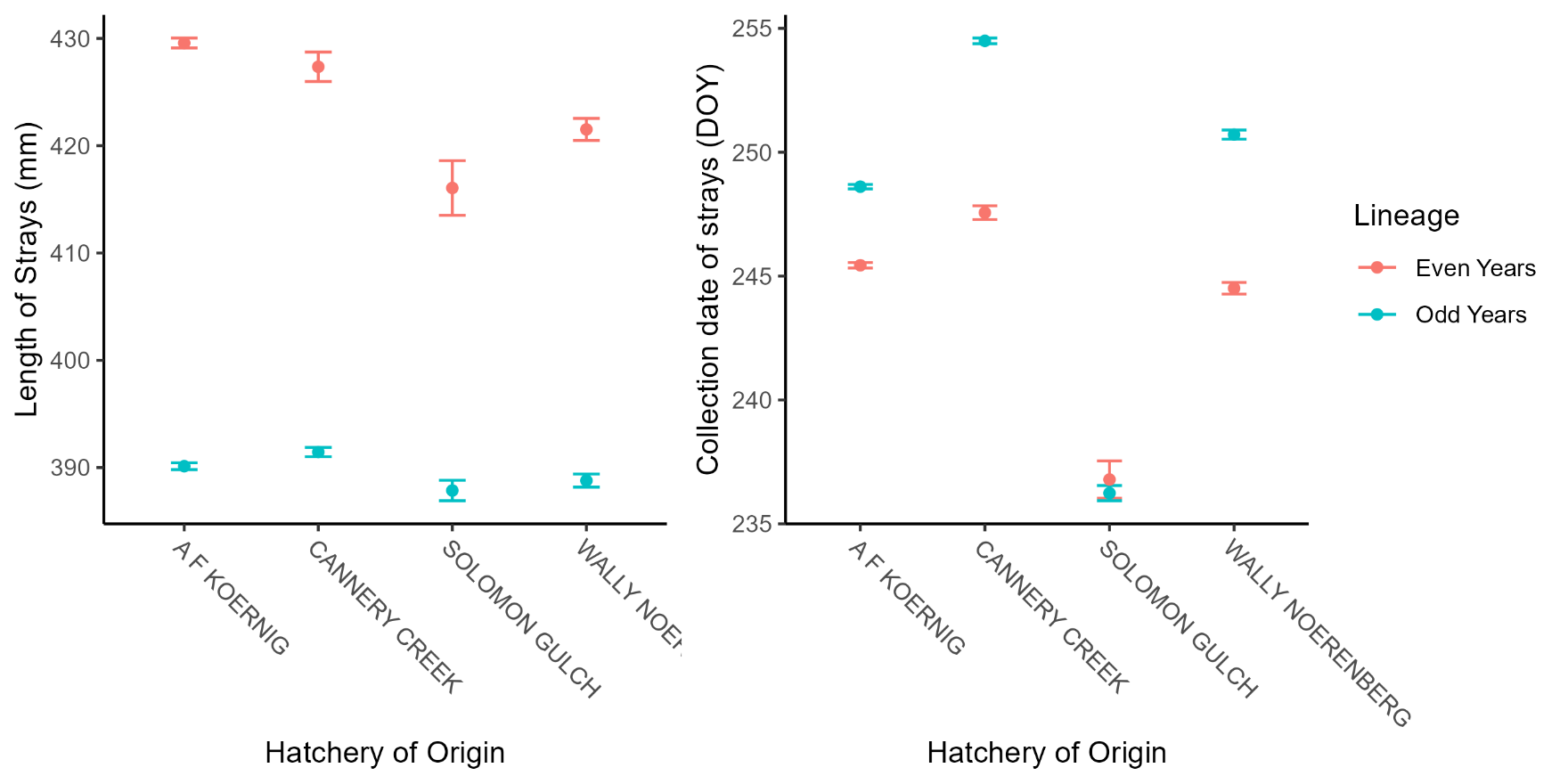


Fig. S3.

Phenotypic differences among adult, hatchery-origin pink salmon sampled in natural streams in PWS, AK. Mean phenotypic values (y-axes) from all strays collected from all streams are provided for body length (mm; left) and collection date (DOY; right). Hatchery of origin (x-axis) was determined using hatchery-specific thermally marked otoliths. Values are colored by lineage, and error bars represent 95% confidence intervals.

Table S1.

Mean mid-eye to hypural-plate body length (mm), standard deviation, and the sample size of otoliths read of hatchery (H) and wild (W) origin sampled from 2013 to 2018. “--” indicates that no hatchery or wild samples were identified at that creek, year, and origin category. Sample location followed by the map number from Figure 1 and the ADF&G anadromous waters catalog number.

| Location | Origin | 2013 | 2014 | 2015 | 2016 | 2017 | 2018 | 2019 | 2020 |
| --- | --- | --- | --- | --- | --- | --- | --- | --- | --- |
| Bainbridge C | H | 395, 25, 25 | -- | 388, 22, 65 | -- | -- | -- | -- | -- |
| (3; 226-20-16300) | W | 404, 30, 219 | 410, 28, 476 | 414, 36, 508 | -- | -- | -- | -- | -- |
| Beartrap R | H | 437, 33, 3 | 419, --, 1 | 424, 31, 5 | -- | -- | -- | -- | -- |
| (16; 221-30-1048) | W | 432, 42, 367 | 413, 30, 459 | 424, 34, 448 | -- | -- | -- | -- | -- |
| Cabin C | H | 392, 20, 11 | 434, 25, 56 | 384, 22, 52 | -- | -- | -- | -- | -- |
| (7; 227-20-17464) | W | 400, 27, 221 | 423, 25, 298 | 393, 28, 489 | -- | -- | -- | -- | -- |
| Coghill R | H | 378, 17, 4 | 448, 27, 24 |  | -- | -- | -- | -- | -- |
| (25; 223-30-13220) | W | 416, 33, 138 | 421, 28, 222 | 429, 34, 461 | -- | -- | -- | -- | -- |
| Comstock C | H | 396, 26, 107 | 417, 24, 441 | 378, 22, 348 | -- | -- | -- | -- | -- |
| (29; 225-20-15040) | W | 394, 33, 11 | 415, 25, 67 | 379, 22, 76 | -- | -- | -- | -- | -- |
| Constantine C | H | -- | 416, 33, 7 | 400, 9, 2 | -- | -- | -- | -- | -- |
| (10; 228-60-18150) | W | 405, 30, 294 | 415, 25, 269 | 406, 32, 528 | -- | -- | -- | -- | -- |
| Double C | H | 400, --, 1 | 408, 34, 7 | 467, 26, 4 | -- | -- | -- | -- | -- |
| (12; 228-40-18310) | W | 414, 36, 574 | 423, 26, 130 | 435, 33, 366 | -- | -- | -- | -- | -- |
| Erb C | H | 394, 24, 43 | 430, 23, 402 | 387, 23, 1967 | 444, 22, 354 | 403, 26, 2267 | 422, 24, 271 | -- | 400, 25, 733 |
| (2; 226-20-16040) | W | 401, 26, 385 | 406, 27, 1087 | 403, 31, 9315 | 434, 24, 3528 | 418, 38, 9901 | 416, 28, 7574 | -- | 406, 27, 6004 |
| Fish C | H | -- | 431, 18, 16 | 388, 16, 11 | -- | -- | -- | -- | -- |
| (19; 221-40-10890) | W | 420, 35, 312 | 419, 27, 414 | 418, 36, 577 | -- | -- | -- | -- | -- |
| Gilmour C | H | -- | 430, 22, 317 | 389, 22, 665 | 434, 22, 137 | 400, 20, 78 | 424, 29, 124 | 381, 24, 856 | -- |
| (8; 227-20-17480) | W | -- | 423, 23, 246 | 387, 26, 2108 | 434, 24, 1018 | 398, 27, 261 | 421, 28, 1045 | 382, 26, 2702 | -- |
| Hartney C | H | 402, 3, 3 | 423, 24, 15 | 395, 25, 5 | -- | -- | -- | -- | -- |
| (13; 221-10-10020) | W | 400, 25, 436 | 432, 27, 203 | 414, 39, 526 | -- | -- | -- | -- | -- |
| Hogan Bay C | H | 405, 22, 426 | 440, 23, 2253 | 385, 23, 4806 | 441, 22, 2445 | 396, 23, 10927 | 420, 25, 5313 | 387, 25, 13212 | -- |
| (5; 226-30-16810) | W | 400, 26, 263 | 426, 27, 233 | 395, 31, 4128 | 437, 22, 9695 | 398, 32, 6204 | 417, 26, 7812 | 386, 28, 4495 | -- |
| Hummer C | H | 414, 16, 2 | 432, 21, 81 | 387, 19, 36 | -- | -- | -- | -- | -- |
| (26; 224-10-14240) | W | 403, 30, 128 | 411, 25, 308 | 413, 36, 383 | -- | -- | -- | -- | -- |
| Humpback C (221-10-10110) | W | -- | 411, 16, 46 | -- | -- | -- | -- | -- | -- |
| Johnson C | H | 393, 26, 43 | 431, 22, 330 | 391, 24, 157 | -- | -- | -- | -- | -- |
| (4; 226-40-16269) | W | 399, 24, 95 | 412, 23, 155 | 408, 32, 448 | -- | -- | -- | -- | -- |
| Lagoon C | H | 391, 17, 5 | 428, 32, 23 | 415, 31, 16 | -- | -- | -- | -- | -- |
| (20; 221-40-10990) | W | 408, 32, 468 | 420, 26, 390 | 425, 36, 545 | -- | -- | -- | -- | -- |
| Long C | H | 401, 8, 3 | 431, 24, 71 | 400, 31, 30 | -- | -- | -- | -- | -- |
| (21; 222-10-12140) | W | 413, 32, 89 | 415, 21, 88 | 430, 35, 400 | -- | -- | -- | -- | -- |
| Paddy C | H | 410, 26, 8 | 430, 23, 555 | 386, 22, 2707 | 444, 20, 157 | 400, 27, 1234 | 413, 27, 104 | -- | 401, 24, 841 |
| (3; 226-20-16010) | W | 391, 22, 70 | 415, 27, 339 | 396, 29, 5577 | 438, 22, 700 | 413, 36, 4397 | 416, 28, 1991 | -- | 407, 26, 4934 |
| Paulson C | H | 393, 28, 18 | 401, 18, 3 | 397, 23, 63 | -- | -- | -- | -- | -- |
| (27; 224-10-14550) | W | 399, 28, 260 | 405, 28, 455 | 418, 35, 473 | -- | -- | -- | -- | -- |
| Sheep R | H | -- | 398, 24, 6 | 415, 23, 2 | -- | -- | -- | -- | -- |
| (15; 221-20-10360) | W | 427, 38, 695 | 417, 30, 417 | 433, 35, 554 | -- | -- | -- | -- | -- |
| Short C | H | 402, 31, 7 | 419, 29, 30 | 403, 25, 14 | -- | -- | -- | -- | -- |
| (18; 221-40-10880) | W | 411, 31, 1093 | 415, 26, 306 | 417, 33, 436 | -- | -- | -- | -- | -- |
| Siwash R | H | 414, 24, 16 | 437, 20, 151 | 390, 25, 118 | -- | -- | -- | -- | -- |
| (24; 222-20-12640) | W | 409, 29, 138 | 414, 28, 302 | 421, 33, 463 | -- | -- | -- | -- | -- |
| Snug Harbor C | H | -- | -- | 393, 24, 25 | -- | -- | -- | -- | -- |
| (11; 226-30-16820) | W | -- | -- | 406, 30, 265 | -- | -- | -- | -- | -- |
| Spring C | H | -- | 415, 12, 5 | 401, 33, 14 | -- | -- | -- | -- | -- |
| (22; 222-10-12170) | W | 391, 28, 67 | 405, 24, 294 | 408, 32, 570 | -- | -- | -- | -- | -- |
| Spring C | H | 387, 34, 26 | 415, --, 1 | 372, 9, 2 | -- | -- | -- | -- | -- |
| (14; 221-20-10200) | W | 400, 31, 1224 | 419, 22, 93 | 401, 32, 376 | -- | -- | -- | -- | -- |
| Stockdale C | H | 395, 21, 157 | 434, 23, 895 | 385, 24, 1656 | 442, 22, 583 | 400, 25, 1036 | 426, 27, 219 | 381, 25, 1290 | -- |
| (9; 227-20-17520) | W | 392, 22, 851 | 420, 27, 395 | 388, 29, 6655 | 439, 25, 7208 | 411, 32, 9617 | 420, 27, 4372 | 384, 27, 6955 | -- |
| Sunny R | H | -- | 409, 33, 9 | 377, 43, 6 | -- | -- | -- | -- | -- |
| (17; 221-40-10875) | W | 419, 35, 260 | 428, 27, 295 | 419, 36, 378 | -- | -- | -- | -- | -- |
| Surplus C | H | -- | 427, 21, 51 | 383, 22, 34 | -- | -- | -- | -- | -- |
| (23; 222-20-12338) | W | 419, 35, 371 | 410, 21, 119 | 433, 35, 447 | -- | -- | -- | -- | -- |
| Swamp C | H | 397, 26, 12 | 436, 30, 21 | 384, 32, 55 | -- | -- | -- | -- | -- |
| (6; 227-20-17390) | W | 397, 27, 259 | 428, 27, 187 | 429, 36, 526 | -- | -- | -- | -- | -- |
| West Finger C | H | 387, 15, 7 | -- | 389, 22, 12 | -- | -- | -- | -- | -- |
| (28; 224-40-14850) | W | 397, 24, 220 | 411, 26, 448 | 412, 36, 389 | -- | -- | -- | -- | -- |

Table S2.

Results of stepwise generalized linear model selection procedures. Models were evaluated with the second-order Akaike Information Criterion (AICc). All models were compared to the best-fit model by comparing AICc values (ΔAICc). The weighted AIC score (wAICc) is the proportion of the total predictive power provided by the full set of models. Log-likelihood (LL) accounts for the number of model parameters (K; degrees of freedom). The null model refers to an intercept-only model with no covariates. Response variables are provided for different suites of sub-models, alongside the data subset used in that mode (i.e., even or odd years).

| **Response** | **Model** | **K** | **LL** | **AICc** | **ΔAICc** | ***w*AICc** |
| --- | --- | --- | --- | --- | --- | --- |
| Even Length | DOY + Origin + Sex + (1 \| Stream) + (1 \| Year) | 7 | -380581 | 761176.1 | 0 | 0.42 |
|  | DOY + Origin + Sex + (1 \| Stream) + (1 \| Year) + Origin:Sex | 8 | -380581 | 761177 | 0.9 | 0.27 |
|  | DOY + Origin + Sex + (1 \| Stream) + (1 \| Year) + DOY:Sex | 8 | -380581 | 761178.1 | 1.92 | 0.16 |
|  | DOY + Origin + Sex + (1 \| Stream) + (1 \| Year) + DOY:Sex + Origin:Sex | 9 | -380580 | 761178.5 | 2.36 | 0.13 |
|  | DOY + Origin + (1 \| Stream) + (1 \| Year) | 6 | -380586 | 761183.2 | 7.08 | 0.01 |
|  | Origin + Sex + (1 \| Stream) + (1 \| Year) | 6 | -380586 | 761183.5 | 7.37 | 0.01 |
|  | Origin + Sex + (1 \| Stream) + (1 \| Year) + Origin:Sex | 7 | -380585 | 761184.3 | 8.14 | 0.01 |
|  | Origin + (1 \| Stream) + (1 \| Year) | 5 | -380591 | 761191.7 | 15.58 | 0 |
|  | Sex + (1 \| Stream) + (1 \| Year) | 5 | -380774 | 761558.5 | 382.39 | 0 |
|  | DOY + Sex + (1 \| Stream) + (1 \| Year) | 6 | -380775 | 761561 | 384.86 | 0 |
|  | DOY + Sex + (1 \| Stream) + (1 \| Year) + DOY:Sex | 7 | -380774 | 761562.1 | 385.91 | 0 |
|  | NULL | 4 | -380780 | 761567.5 | 391.37 | 0 |
|  | DOY + (1 \| Stream) + (1 \| Year) | 5 | -380780 | 761569.5 | 393.38 | 0 |
| Odd Length | DOY + Origin + Sex + (1 \| Stream) + (1 \| Year) + DOY:Sex + Origin:Sex | 9 | -655065 | 1310148 | 0 | 1 |
|  | DOY + Origin + Sex + (1 \| Stream) + (1 \| Year) + DOY:Sex | 8 | -655106 | 1310228 | 79.85 | 0 |
|  | DOY + Sex + (1 \| Stream) + (1 \| Year) + DOY:Sex | 7 | -655244 | 1310502 | 353.86 | 0 |
|  | DOY + Origin + Sex + (1 \| Stream) + (1 \| Year) + Origin:Sex | 8 | -655497 | 1311010 | 862.14 | 0 |
|  | DOY + Origin + Sex + (1 \| Stream) + (1 \| Year) | 7 | -655690 | 1311394 | 1246.16 | 0 |
|  | DOY + Sex + (1 \| Stream) + (1 \| Year) | 6 | -655832 | 1311676 | 1527.9 | 0 |
|  | DOY + Origin + (1 \| Stream) + (1 \| Year) | 6 | -655833 | 1311678 | 1529.6 | 0 |
|  | DOY + (1 \| Stream) + (1 \| Year) | 5 | -655978 | 1311966 | 1817.97 | 0 |
|  | Origin + Sex + (1 \| Stream) + (1 \| Year) + Origin:Sex | 7 | -659435 | 1318885 | 8736.45 | 0 |
|  | Origin + Sex + (1 \| Stream) + (1 \| Year) | 6 | -659669 | 1319350 | 9201.37 | 0 |
|  | Origin + (1 \| Stream) + (1 \| Year) | 5 | -659827 | 1319664 | 9515.62 | 0 |
|  | Sex + (1 \| Stream) + (1 \| Year) | 5 | -660219 | 1320447 | 10298.79 | 0 |
|  | NULL | 4 | -79837 | 1320778 | 10629.21 | 0 |
| Even Timing | Origin + Sex + (1 \| Stream) + (1 \| Year) + Origin:Sex | 7 | -79839 | 159688 | 0 | 0.96 |
|  | Length + Origin + Sex + (1 \| Stream) + (1 \| Year) + Origin:Sex | 8 | -79846 | 159694.2 | 6.16 | 0.04 |
|  | Length + Origin + Sex + (1 \| Stream) + (1 \| Year) + Length:Sex + Origin:Sex | 9 | -79850 | 159709 | 21.01 | 0 |
|  | Origin + Sex + (1 \| Stream) + (1 \| Year) | 6 | -79852 | 159712.2 | 24.22 | 0 |
|  | Length + Origin + Sex + (1 \| Stream) + (1 \| Year) | 7 | -79858 | 159718.2 | 30.24 | 0 |
|  | Length + Origin + Sex + (1 \| Stream) + (1 \| Year) + Length:Sex | 8 | -79943 | 159731.3 | 43.34 | 0 |
|  | Origin + (1 \| Stream) + (1 \| Year) | 5 | -79945 | 159896.6 | 208.64 | 0 |
|  | Length + Origin + (1 \| Stream) + (1 \| Year) | 6 | -80314 | 159901.5 | 213.47 | 0 |
|  | Sex + (1 \| Stream) + (1 \| Year) | 5 | -80322 | 160638.8 | 950.83 | 0 |
|  | Length + Sex + (1 \| Stream) + (1 \| Year) | 6 | -80327 | 160655.4 | 967.44 | 0 |
|  | Length + Sex + (1 \| Stream) + (1 \| Year) + Length:Sex | 7 | -80404 | 160668.2 | 980.15 | 0 |
|  | NULL | 4 | -80411 | 160816 | 1127.98 | 0 |
|  | Length + (1 \| Stream) + (1 \| Year) | 5 | -79837 | 160832.1 | 1144.06 | 0 |
| Odd Timing | Length + Origin + Sex + (1 \| Stream) + (1 \| Year) + Length:Sex + Origin:Sex | 9 | -173163 | 346343.5 | 0 | 1 |
|  | Length + Origin + Sex + (1 \| Stream) + (1 \| Year) + Origin:Sex | 8 | -173177 | 346369.1 | 25.54 | 0 |
|  | Length + Origin + Sex + (1 \| Stream) + (1 \| Year) + Length:Sex | 8 | -173181 | 346378.8 | 35.23 | 0 |
|  | Length + Origin + (1 \| Stream) + (1 \| Year) | 6 | -173186 | 346383.4 | 39.84 | 0 |
|  | Length + Origin + Sex + (1 \| Stream) + (1 \| Year) | 7 | -173189 | 346391.9 | 48.32 | 0 |
|  | Length + Sex + (1 \| Stream) + (1 \| Year) + Length:Sex | 7 | -175157 | 350327.3 | 3983.73 | 0 |
|  | Length + (1 \| Stream) + (1 \| Year) | 5 | -175165 | 350339.6 | 3996.06 | 0 |
|  | Length + Sex + (1 \| Stream) + (1 \| Year) | 6 | -175167 | 350345.5 | 4001.92 | 0 |
|  | Origin + Sex + (1 \| Stream) + (1 \| Year) + Origin:Sex | 7 | -177099 | 354212.4 | 7868.83 | 0 |
|  | Origin + Sex + (1 \| Stream) + (1 \| Year) | 6 | -177152 | 354315.9 | 7972.34 | 0 |
|  | Origin + (1 \| Stream) + (1 \| Year) | 5 | -177164 | 354338.1 | 7994.51 | 0 |
|  | Sex + (1 \| Stream) + (1 \| Year) | 5 | -179537 | 359083.6 | 12740.06 | 0 |
|  | NULL | 4 | -179555 | 359117.9 | 12774.4 | 0 |
| Difference in Even Year Mean Hatchery Length (PWS - Stream) | NULL | 3 | -142 | 291.2 | 0 | 0.70 |
|  | Difference in Mean Wild Length (PWS - Stream) | 4 | -142 | 292.9 | 1.66 | 0.30 |
| Difference in Odd-Year Mean Hatchery Length (PWS - Stream) | Difference in Mean Wild Length (PWS - Stream) | 4 | -225 | 459.8 | 0 | 1 |
|  | NULL | 3 | -234 | 474.6 | 14.86 | 0 |
| Difference in Even-Year Mean Hatchery Return Day (PWS - Stream) | Mean Wild Return Day (PWS - Stream) | 4 | -120 | 251.0 | 0 | 1 |
|  | NULL | 3 | -136 | 280.1 | 29.16 | 0 |
| Difference in Odd-Year Mean Hatchery Return Day (PWS - Stream) | Mean Wild Return Day (PWS - Stream) | 4 | -186 | 380.2 | 0 | 1 |
|  | NULL | 3 | -202 | 410.5 | 30.27 | 0 |
